# Compositional and functional characterisation of biomass-degrading microbial communities in guts of plant fibre- and soil-feeding higher termites

**DOI:** 10.1186/s40168-020-00872-3

**Published:** 2020-06-23

**Authors:** Martyna Marynowska, Xavier Goux, David Sillam-Dussès, Corinne Rouland-Lefèvre, Rashi Halder, Paul Wilmes, Piotr Gawron, Yves Roisin, Philippe Delfosse, Magdalena Calusinska

**Affiliations:** 1 Luxembourg Institute of Science and Technology, 41 rue du Brill, L-4422 Belvaux, Luxembourg; 2 Université Paris 13–Sorbonne Paris Cité, LEEC, EA 4443 Villetaneuse, France; 3 iEES-Paris, Institute of Research for Development, Sorbonne Universités, U 242 Bondy, France; 4 Luxembourg Centre for Systems Biomedicine, University of Luxembourg, 7 avenue des Hauts-Fourneaux, L-4362 Esch-sur-Alzette, Luxembourg; 5 Université Libre de Bruxelles, 50 avenue F.D. Roosevelt, B-1050 Brussels, Belgium; 6 University of Luxembourg, 2 avenue de l’Université, L-4365 Esch-sur-Alzette, Luxembourg

**Keywords:** Termite gut microbiome, Metatranscriptomics, 16S rRNA gene sequencing, Isoptera, CAZymes, Lignocellulose decomposition

## Abstract

**Background:**

Termites are among the most successful insect lineages on the globe and are responsible for providing numerous ecosystem services. They mainly feed on wood and other plant material at different stages of humification. Lignocellulose is often a principal component of such plant diet, and termites largely rely on their symbiotic microbiota and associated enzymes to decompose their food efficiently. While lower termites and their gut flagellates were given larger scientific attention in the past, the gut lignocellulolytic bacteria of higher termites remain less explored. Therefore, in this study, we investigated the structure and function of gut prokaryotic microbiomes from 11 higher termite genera representative of *Syntermitinae*, *Apicotermitinae*, *Termitidae* and *Nasutitermitinae* subfamilies, broadly grouped into plant fibre- and soil-feeding termite categories.

**Results:**

Despite the different compositional structures of the studied termite gut microbiomes, reflecting well the diet and host lineage, we observed a surprisingly high functional congruency between gut metatranscriptomes from both feeding groups. The abundance of transcripts encoding for carbohydrate active enzymes as well as expression and diversity profiles of assigned glycoside hydrolase families were also similar between plant fibre- and soil-feeding termites. Yet, dietary imprints highlighted subtle metabolic differences specific to each feeding category. Roughly, 0.18% of de novo re-constructed gene transcripts were shared between the different termite gut microbiomes, making each termite gut a unique reservoir of genes encoding for potentially industrially applicable enzymes, e.g. relevant to biomass degradation. Taken together, we demonstrated the functional equivalence in microbial populations across different termite hosts.

**Conclusions:**

Our results provide valuable insight into the bacterial component of the termite gut system and significantly expand the inventory of termite prokaryotic genes participating in the deconstruction of plant biomass.

Video Abstract

## Background

Termites are eusocial insects that greatly contribute to the carbon and nitrogen cycling in tropical ecosystems and provide multiple other ecosystem services, e.g. litter decomposition, bioturbation or water infiltration [1]. They mainly feed on plant material in a form of sound wood or at different stages of humification such as leaf litter, humus, and soil organic matter [2]. Lignocellulose, a principal component of plant biomass, is mainly composed of cross-linked cellulose, hemicellulose and lignin which form a structure recalcitrant to enzymatic hydrolysis [3]. Owing to specific adaptations developed over millions of years, the efficiency of lignocellulose decomposition by higher termites exceeds that of many other known natural systems [4], making them desirable models for bioprospecting with emphasis on industrial conversion of lignocellulose and the production of biofuels and other commodity biochemicals.

Despite secreting endogenous enzymes by their midgut epithelium or labial glands [5], termites’ ability to decompose lignocellulose largely depends on the mutualistic symbiosis with diverse gut microorganisms, including bacteria and archaea in the case of the family *Termitidae* (“higher” termites) and flagellate protists in basal lineages (“lower” termites) [6]. Members of the *Macrotermitinae* subfamily are a unique example of higher termites characterised by an additional exo-symbiosis with *Termitomyces* fungi that initially predigest the biomass, subsequently consumed by termites. Of special interest, it is the prokaryotic component of the termite gut system which contributes not only to the digestion of plant fibre, but also to the host nutrition [6].

Similar to many other natural systems, most of the prokaryotes in the termite guts are uncultivable. Through the application of recent culture-independent molecular approaches, in particular the high-throughput sequencing targeting the 16S rRNA gene, the gut microbial diversity and community structure of multiple termite species are now well established. Rampant host-switching was proposed as a model behind the assemblage of microbiota in termite guts [7, 8]. Several studies took advantage of the metagenomics (high-throughput sequencing of the total community DNA) and offered an insight into the metabolic potential of the gut prokaryotic community in higher wood-feeding termites, including *Nasutitermes* spp. [9, 10] and *Globitermes brachycerastes* [11], as well as the dung-feeding termite, *Amitermes wheeleri* [10]. Numerous protein-coding genes relevant to lignocellulose decomposition as well as hydrogen metabolism, nitrogen fixation and reductive acetogenesis have been reported. It is now known that mutualistic symbionts provide their termite host with a set of carbohydrate-active enzymes (CAZymes), including glycoside hydrolases (GH), polysaccharide lyases (PL) and carbohydrate esterases (CE), as well as other enzymes with auxiliary activities (AA), that together contribute to the lignocellulose degradation in the termite gut. Additionally, non-catalytic carbohydrate-binding modules (CBM) promote the association of these enzymes with specific polysaccharide substrates [12, 13]. Another study gave an insight into the organisation of fibrolytic genes by reporting the presence of putative saccharolytic operons and therefore suggesting that clustering of CAZymes is common in termite gut microbiota [11]. By highlighting changes in gene expression profiles, metatranscriptomics (high-throughput whole-community mRNA sequencing) could reveal a much more dynamic picture of the microbial community than metagenomics. Still, metatranscriptomics of higher termite gut microbiomes is only emerging, with roughly three studies published to date, to the best of our knowledge [10, 14, 15]. Already, these limited reports have highlighted the significance of de novo metatranscriptomics to constrain the estimates of particular microbial processes, which otherwise might have been overlooked in the corresponding metagenomic datasets [10].

To further elucidate the strategies of bacterial lignocellulose degradation by the higher termite gut system, especially in regard to different feeding habits of the host, we performed an integrative analysis of bacterial community structure and function, which we applied to 41 higher termite colonies (15 different genera), characterised by diverse diets (including wood, grass, soil, humus, litter, and microepiphytes). First, we analysed gut bacterial communities in all samples by the means of 16S rRNA gene amplicon sequencing. Then, taking advantage of our previously optimised metatranscriptomic framework [14], we performed high-throughput profiling of prokaryotic metatranscriptomes originating from 11 selected higher termite species, giving special attention to genes encoding enzymes relevant to plant biomass deconstruction. As a result, we extended current knowledge on lignocellulose degradation by the higher termite gut system [9, 10] and inferred it to a higher diversity of termite genera feeding on various carbon sources, including plant fibres (e.g. wood and grass) as well as soil, humus and few others. Moreover, we showed that each termite species is a unique organism operating with its own bacterial flora and genes repertoire.

## Methods

### Sample collection and termite species identification

Forty-one higher termite colonies were sampled in March 2016 and January 2017 in the tropical forest and savannah of French Guiana. Fifteen higher termite genera with various feeding habits were targeted (Table 1). They were broadly classified as plant fibre (wood, grass, and microepiphytes) and soil (soil, humus, and litter) feeders. Mature workers retrieved from the nests were cold immobilised, surface-cleaned with 80 % ethanol and 1 × PBS and decapitated. Whole guts were dissected (*n* ≈ 30 guts per replicate, minimum three replicates per sample) and preserved directly in either liquid nitrogen or RNAlater Stabilization Solution (Ambion). Additionally, for 11 selected samples (to be further tested by metatranscriptomic approach, see Table 1), the luminal fluid was collected as previously described, in order to reduce the amount of host tissue contamination [14]. Samples were stored at − 80 °C until further processing. The termite species were identified by morphology and by sequencing of the partial CO-II (cytochrome oxidase subunit 2) marker gene. To that purpose, DNA was extracted from termite heads using AllPrep DNA/RNA Micro Kit (Qiagen) following manufacturer’s protocol, supplemented by bead-beating (2 × 5 mm and 5 × 2 mm sterile milling beads) at 15 Hz for 2 min. The analysis of CO-II sequences was performed using A-tLeu and B-tLys primers, as previously described [19]. The nucleotide sequences are available in GenBank under accession numbers from MH067978 to MH068018.

**Table 1:** Overview of the higher termite species included in this study

Sample	Species^**a**^	Subfamily	Feeding habit^**b**^	Metatranscriptomics
“Plant fibre feeders” [sound lignocellulose]				
N.sim_1	*Nasutitermes similis*	*Nasutitermitinae*	Wood	
N.sim_2	*Nasutitermes similis*	*Nasutitermitinae*	Wood	
N.sim_3	*Nasutitermes similis*	*Nasutitermitinae*	Wood	
N.sim_4	*Nasutitermes similis*	*Nasutitermitinae*	Wood	+
N.sp_1	*Nasutitermes* sp.	*Nasutitermitinae*	Wood	
N.eph_1	*Nasutitemes ephratae*	*Nasutitermitinae*	Wood	
N.eph_2	*Nasutitemes ephratae*	*Nasutitermitinae*	Wood	
N.eph_3	*Nasutitemes ephratae*	*Nasutitermitinae*	Wood	
N.eph_4	*Nasutitemes ephratae*	*Nasutitermitinae*	Wood	
C.int	*Cortaritermes intermedius*	*Nasutitermitinae*	Litter/grass	
N.oct	*Nasutitermes octopilis*	*Nasutitermitinae*	Wood	
N.sp_2	*Nasutitermes* sp.	*Nasutitermitinae*	Wood	+
C.cav	*Constrictotermes cavifrons*	*Nasutitermitinae*	Microepiphytes/wood	+
M.sp_1	*Microcerotermes* sp.	*Termitinae*	Wood	
M.sp_2	*Microcerotermes* sp.	*Termitinae*	Wood	+
“Soil feeders” [humified lignocellulose]				
C.pug	*Cornitermes pugnax*	*Syntermitinae*	Litter/decayed wood	+
C.tub_1	*Cavitermes tuberosus*	*Termitinae*	Humus	
C.tub_2	*Cavitermes tuberosus*	*Termitinae*	Humus	
C.tub_3	*Cavitermes tuberosus*	*Termitinae*	Humus	
L.lab_1	*Labiotermes labralis*	*Termitinae*	Soil	
L.lab_2	*Labiotermes labralis*	*Termitinae*	Soil	
L.lab_3	*Labiotermes labralis*	*Termitinae*	Soil	
L.lab_4	*Labiotermes labralis*	*Termitinae*	Soil	+
E.neo_1	*Embiratermes neotenicus*	*Syntermitinae*	Humus	+
E.neo_2	*Embiratermes neotenicus*	*Syntermitinae*	Humus	
E.neo_3	*Embiratermes neotenicus*	*Syntermitinae*	Humus	
C.ang	*Cyrilliotermes angulariceps*	*Syntermitinae*	Humus	+
S.hey_1	*Silvestritermes heyeri*	*Syntermitinae*	Soil	+
S.hey_2	*Silvestritermes heyeri*	*Syntermitinae*	Soil	
S.hey_3	*Silvestritermes heyeri*	*Syntermitinae*	Soil	
S.hey_4	*Silvestritermes heyeri*	*Syntermitinae*	Soil	
S.min	*Silvestritermes minutus*	*Syntermitinae*	Soil	
A.cin	*Aparatermes cingulatus*	*Apicotermitinae*	Humus	
A.ban_1	*Anoplotermes banksi*	*Apicotermitinae*	Humus	
A.ban_2	*Anoplotermes banksi*	*Apicotermitinae*	Humus	+
N.tar_1	*Neocapritermes taracua*	*Termitinae*	Humus	+
N.tar_2	*Neocapritermes taracua*	*Termitinae*	Humus	
T.fat_1	*Termes fatalis*	*Termitinae*	Humus	
T.fat_2	*Termes fatalis*	*Termitinae*	Humus	
T.fat_3	*Termes fatalis*	*Termitinae*	Humus	
S.sp	*Syntermes* sp.	*Syntermitinae*	Litter	

^a^Species were classified based on the closest match identified in the NCBI repository

^b^Feeding habit was defined following [16–18]

### Nucleic acid extraction

DNA and RNA from whole guts and luminal fluid were co-extracted from all samples using the AllPrep PowerViral DNA/RNA Kit (Qiagen) following manufacturer’s protocol. To guarantee the proper disruption of bacterial cells, the mechanical bead-beating step with 0.1 mm glass beads at 20 Hz for 2 min was introduced to complement the chemical lysis. The eluents were divided in half. The first aliquot was treated with 1 μL of 10 μg/ml RNase A (Sigma) for 30 min at room temperature. The second one was treated with TURBO DNA-free kit (Invitrogen) according to manufacturer’s protocol. DNAse Inactivation reagent step in purification of RNA was replaced by Agencourt RNAClean XP Kit purification step (Beckman Coulter). The resulting pure DNA and RNA were quality assessed using agarose gel electrophoresis and Bioanalyser RNA 6000 Pico Kit (Agilent). The concentration was quantified using Qubit dsDNA HS Assay and Qubit RNA HS Assay Kit (Invitrogen). DNA and RNA were stored at − 20 °C and − 80 °C, respectively.

### Bacterial 16S rRNA gene amplicon sequencing and analysis

The bacterial 16S rRNA gene amplicon libraries for all 41 whole guts samples were prepared using Illumina compatible approach as previously described [14]. Briefly, modified universal primers S-D-Bact-0909-a-S-18 and S-*-Univ-*-1392-a-A-15 [20] and Nextera XT Index Kit V2 (Illumina) were used along with Q5 Hot Start High-Fidelity 2× Master Mix (New England Biolabs) to perform two-step PCR. It allowed for selective amplification of the 484-bp long fragment of bacterial 16S rRNA gene V6–V8 region and simultaneous attachment of Illumina adapters and barcodes. Negative control with no DNA template was included in each PCR reaction to assess any possible contamination. Purified and equimolarly pooled libraries were sequenced along with PhiX control (Illumina) using MiSeq Reagent Kit V3-600 on in-house Illumina Mi-Seq Platform. The CLC Genomics Workbench v.9.5.2 and Usearch v.7.0.1090_win64 software [21] were used for quality trimming, chimera check, singletons removal and assignment of the obtained sequences to operational taxonomic units (OTUs) at 97% similarity level. Taxonomic affiliation of the resulting OTUs was performed with DictDB database [22] using *mothur* [23]*.* Due to the good correlation of triplicates (Additional file 1: Figure S1), reads for biological replicates were pooled and re-analysed together. The sequencing reads are available in the Sequence Read Archive (SRA) database under accession number SRP135739. Further diversity analysis were performed on the normalised reads (rarefied to 10,000) of bacterial origin, using *mothur* [23] and R environment [24]. Bacterial community richness and diversity were calculated using *sobs* and *invsimpson* calculators, respectively. The structure and membership of bacterial communities between samples were compared using Bray-Curtis dissimilarity and Jaccard similarity indexes. Statistical significance of the results was calculated using ANOSIM, and the differences were considered statistically significant at *p* value ≤ 0.05. The influence of the feeding habit (sound and humified lignocellulose) and taxonomy of the host (genus/subfamily) were tested with PERMANOVA on Bray-Curtis distance matrices (adonis2 function in R library vegan [25];). Furthermore, similarities in the structure of the communities, incorporating phylogenetic distances between observed organisms (OTUs,) were determined using weighted and unweighted UniFrac metric [26] implemented in *mothur*. As a perquisite, the multiple alignment of the OTUs was performed using MUSCLE [27] and refined using MaxAlign [28]. A maximum-likelihood tree was constructed using FastTree2 [29]. Pairwise distances between all samples obtained from UniFrac were then ordinated using NMDS.

### Prokaryotic mRNA sequencing and data analysis

For 11 selected samples, the de novo metatranscriptomic analysis was performed using an optimised approach described previously [14]. Since earlier studies using similar approaches reported a good correlation between the replicates [30, 31], we decided to analyse a broader number of termite species with different feeding habits, at the expense of replicates. Still, for two selected colonies (E.neo_1, S.hey_1), duplicates of metatranscriptomic libraries were prepared to verify the reproducibility of generated results (Additional file 1: Figure S2). The combination of Ribo-Zero Gold rRNA Removal Kit “Epidemiology” (Illumina) and Poly(A)Purist MAG KIT (Ambion) was used to enrich the sample for prokaryotic mRNA. Enriched mRNA was purified using Agencourt RNAClean XP Kit and analysed with Bioanalyser RNA 6000 Pico Kit (Agilent). In continuation, SMARTer Stranded RNA-Seq Kit (Clontech) was used according to the manufacturer’s instructions to prepare metatranscriptomic libraries, using the enriched prokaryotic mRNA as input. The resulting libraries were quantified with High Sensitivity DNA Kit (Agilent) and KAPA SYBR FAST Universal qPCR Kit. Size distribution of the libraries ranged between 331 and 525 bp, with the average of 415 bp. Libraries were pair-end sequenced using Illumina NextSeq 500/550 Mid Output and High Output v2-300 Kits. Raw sequencing reads are available in the SRA database under the accession number SRP135739. Raw reads were quality trimmed in CLC Genomics Workbench v.9.5.2, using a phred quality score of 20, minimum length of 50, removal of 3 nt at 5’ end and allowing no ambiguous nucleotides. Contaminating rRNA reads were further removed using the SortMeRNA 2.0 software [32]. The resulting non-rRNA reads were used to perform de novo metatranscriptomic co-assembly using the CLC assembly algorithm in mapping mode with default parameters except for minimum contig length of 200, length fraction of 0.90 and similarity fraction 0.95. Obtained contigs were further submitted to IMG-MER for open reading frames (ORFs) prediction as well as taxonomic and functional annotation [33]. Following the taxonomic assignment, transcripts of putative prokaryotic origin were selected for further analysis. To improve the taxonomic classification, transcripts were also compared to the metagenome assembled genomes (MAGs) reconstructed in metagenomic study of higher termites gut microbiota [34]. In the case where the identity to MAGs was higher than to the entries in IMG-MER genomes database, the initial IMG-MER taxonomy was corrected. Transcripts encoding for CAZymes were searched with the dbCAN (dbCAN-fam-HMMs.txt.v6) [35] against a CAZy database [12]. Using the thresholds (*e* value of < 10^−18^ and coverage > 0.35) recommended for prokaryotic CAZymes search resulted in removal of a high number of false negatives, therefore, all the genes with annotation to CAZy database were retained and further analysed, keeping in mind that some of them might be false positives. Both results with and without the threshold are presented for comparison purposes. Additionally, the transcripts putatively encoding for CAZymes were further given an enzyme commission number (EC) using homology search to peptide pattern (Hotpep) [36]. In order to determine the relative abundance of all the transcripts across studied samples, the trimmed and filtered sequencing reads were mapped back to the prokaryotic transcripts set, using the CLC “RNA-seq analysis” mode, with default parameters except for minimum similarity of 0.95 over 0.9 of the read length, both strands specificity and one maximum number of hits per read. The mapping results were represented as TPMs (transcripts per million) [37] what directly resulted in normalised read counts. The detailed summary of mRNA sequencing results is presented in Additional file 2: Table S1.

## Results and discussion

### Compositional structure of bacterial microbiomes in higher termite gut reflects the diet and lineage of the host

According to the recent reports, the host diet appears to be the major determinant of the bacterial community structure in higher termite guts [38], and the dietary changes in the feeding routine affect the composition of gut microbiota [39]. Still, the importance of the host signal and previous indications of vertical inheritance [40] should not be neglected. To characterise the diversity of microbial communities associated with the termite gut, we analysed 41 gut samples collected from workers of 15 different termite genera with distinctive feeding habits. Thus, we extended the currently existing knowledge to gut bacterial communities from several previously understudied higher termite species from *Syntermitinae*, *Apicotermitinae*, *Termitidae* and *Nasutitermitinae* subfamilies. The high-throughput sequencing of bacterial 16S rRNA gene amplicons resulted in 4,086,163 reads, further rarefied to 10,000 reads per library, and assigned to 8,069 bacterial OTUs (defined at 97% sequence similarity, Additional file 2: Table S2). The calculated rarefaction curves inferred from species richness reached a plateau, except for a few more diverse samples from soil-feeding termites (Additional file 1: Figure S3). As inferred from Boneh estimate, increased sequencing depth would have allowed describing on average 165 ± 79 additional bacterial OTUs.

**Fig. 1:**
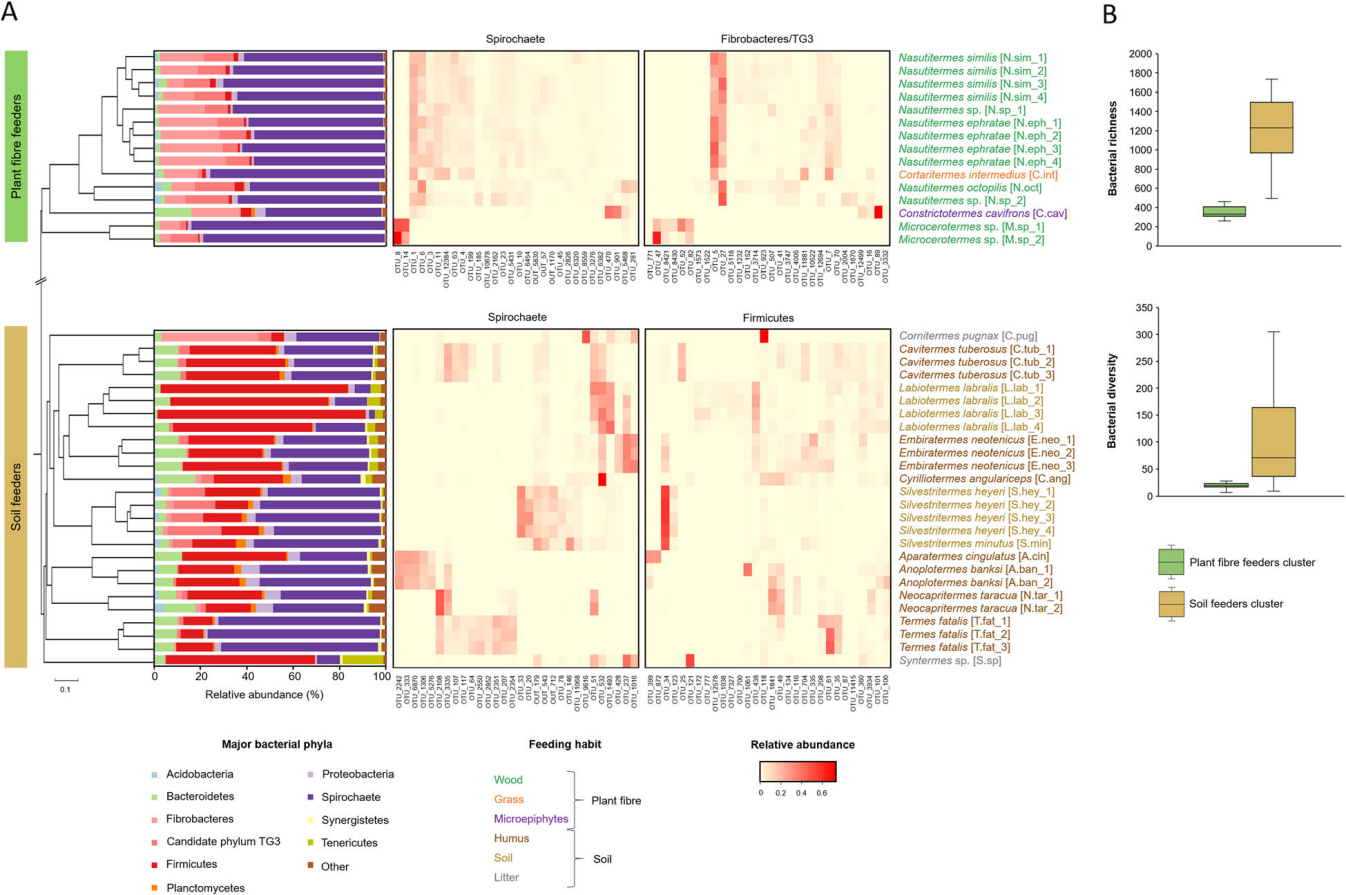
Taxonomic profiles of 41 termite gut bacterial communities. **a** Tree based on the calculated Bray-Curtis dissimilarity in bacterial community structure based on 16S rRNA gene amplicon sequencing, together with the distribution of OTUs into major bacterial phyla and heat map representation of relative abundance of dominant OTUs from major phyla in plant fibre- and soil-feeding termite clusters. ANOSIM R was equal to 0.91 with *p* < 0.001. **b** Box plot representation of average richness (number of observed OTUs inferred using *sobs* calculator) and diversity (inferred using the inverse of the Simpson diversity estimator) of termite gut bacterial communities in plant fibre and soil feeder clusters

To simplify the comparative analyses, the studied gut microbiomes were classified into two broad groups, based on their diets: (a) the “plant fibre feeders”, relying on sound lignocellulose sources including wood, grass and microepiphytes, and (b) the “soil feeders”, relaying on more humified lignocellulose such as soil, humus and litter (Table 1). Following the analysis of dissimilarity in community structure (Bray-Curtis) and membership (Jaccard) at the OTU level, termite gut microbiomes clustered (according to known host dietary preferences (Fig. 1a, Additional file 1: Figures S4-S5). ANOSIM R was equal to 0.91 and 0.98 (*p* < 0.001) for Bray-Curtis and Jaccard, respectively. Similar clustering pattern was also obtained using the weighted and unweighted UniFrac analyses (Additional file 1: Figure S6), which in addition take into account phylogenetic distances between observed organisms (OTUs) [26]. In agreement with a previous report [10], the gut microbiomes of the plant fibre-feeding termites were characterised with an average microbial richness and diversity indices three to five times lower (richness 347 ± 61 and diversity 19 ± 6) than those of the soil-feeding termites (richness 1212 ± 326 and diversity 102 ± 83, Fig. 1b). For plant fibre feeders, roughly 31.3 ± 11.8 of top abundant OTUs represented 80% of reads abundance in a sample, while the same was represented by 247.7 ± 134.4 OTUs for soil feeders. Following the taxonomic annotation of the resulting bacterial OTUs, 26 bacterial phyla were identified (Additional file 2: Table S2). The patterns of bacterial community compositions were consistent with those reported previously for hosts with similar feeding strategies [38]. Unlike for the plant fibre feeders, the taxonomic profiles of the soil-feeding higher termite gut microbiomes were more heterogeneous between the termite species, most probably following the humification (decomposition) gradient of the diet. On average, the plant fibre-feeding termite cluster was dominated by *Spirochaetae* (63.9% ± 9.1 relative community abundance), *Fibrobacteres* (16.6% ± 7.7) and candidate phylum TG3 (10.0% ± 5.2, Fig. 1a). By contrast, *Spirochaetae* was much less abundant in soil feeders (38.4 % ± 18.4), followed by *Firmicutes* (34.8 % ± 22.2) and *Bacteroidetes* (7.6% ± 3.9 reads).

Our results also demonstrated that occurrence and abundance of specific OTUs assigned to the same bacterial phylum differed strongly depending on the termite lineage, regardless of the feeding habit of the termite host (Fig. 1a). For example, at the termite genus level, we could find that highly abundant OTU_5 and OTU_27 assigned to the *Fibrobacteres* phylum were enriched in *Nasutitermes*-specific subcluster, whereas OTU_82 and OTU_771 (also *Fibrobacteres*) were characteristic to *Microcerotermes* sp. Within soil feeders, the examples included preferential association of OTU_428 (*Spirochaetes*) with *Embiratermes* sp., and OTU_20 and OTU_33 being highly abundant mostly in *Silvestritermes* sp. Interestingly, none of the OTUs was shared among all of the 41 studied samples. The PERMANOVA analysis (*p* < 0.001) on the Bray-Curtis distance matrices for bacterial community profiles confirmed that the host feeding regime and the host taxonomy were two important factors shaping the gut bacterial community. Our observations thus extended previous reports to a broader number of termite species [38, 41]. It is important to note that within the same or similar feeding habits, clear compositional divergence was observed for the studied microbiomes at higher taxonomic resolution, where the occurrence and abundance of certain OTUs assigned to the same phyla were strongly dependent on the termite taxonomy. It is in line with recent studies [7, 8], which already postulated the presence of bacterial lineages specific to particular termite groups and suggested the mixed-mode transmission mechanisms (colony-to-offspring and colony-to-colony) of gut bacteria between termites.

### Transcripts annotation to broad functional categories reveals shared metabolic signatures between plant fibre- and soil-feeding termite gut symbionts

**Fig. 2:**
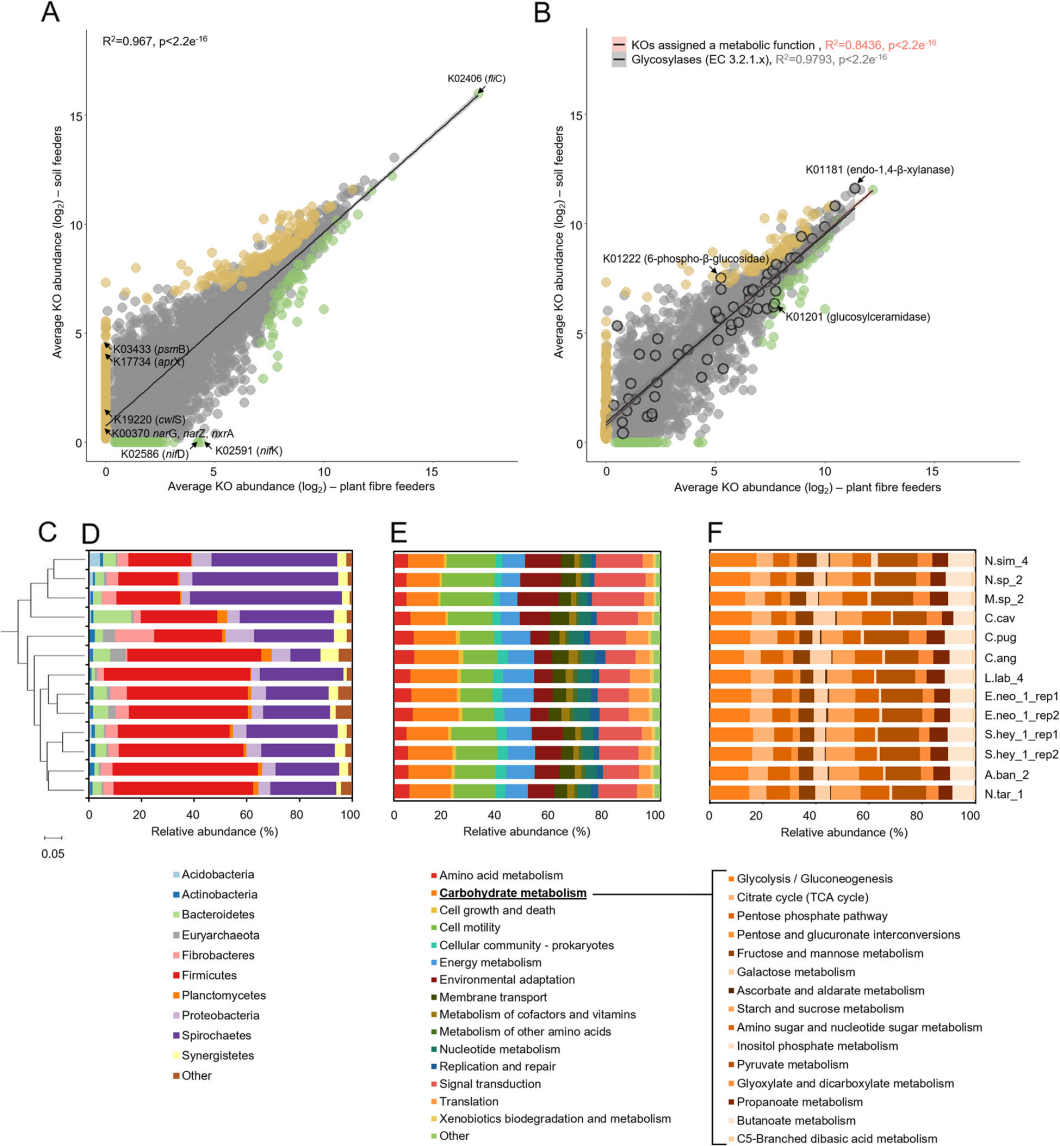
Functional congruency between the soil and the plant fibre feeder gut metatranscriptomes**. a** Average cumulative expression of all transcripts annotated to KEGG Ontology categories, for plant fibre- and soil-feeding termite clusters. Transcripts enriched (according to LEfSe analysis [41]) or present exclusively in plant fibre- or soil-feeding termite cluster are marked in green and brown colour, respectively. *psm*B, proteasome β-subunit; *apr*X, serine protease; *cwl*S, peptidoglycan DL-endopeptidase**;***nar*G/*nar*Z/*nxr*A, nitrate reductase/nitrite oxidoreductase α-subunit; *nif*D and *nif*K, nitrogenase molybdenum-iron protein chains. **b** Average cumulative expression of all transcripts with predicted metabolic activity (based on annotation to BRITE database), for plant fibre- or soil-feeding termite clusters. Transcripts annotated to enzyme class EC.3.2.1.x (glycosylases) are marked with black frame around the dot. Transcripts enriched or present exclusively in plant fibre- or soil- feeding termite cluster are marked in green and brown colour, respectively. **c** Tree based on calculated Bray-Curtis dissimilarity of prokaryotic metatranscriptomic profiles. **d** Putative taxonomic origin of prokaryotic gene transcripts with KO assignment. Relative abundances of phyla were derived from number of sequencing reads mapped to the de novo re-constructed transcripts. **e** Major metabolic pathway categories identified in 11 tested prokaryotic microbiomes. For colonies E.neo_1 and S.hey_1, results for the two replicates are presented. **f** Modules related to carbohydrate metabolism identified in 11 tested prokaryotic microbiomes. For colonies E.neo_1 and S.hey_1, results for the two replicates are presented

Following the community structure analysis (Fig. 1), selected samples representative of 11 different termite species were further studied by sequencing of the enriched prokaryotic mRNA. De novo metatranscriptomic approach was chosen over the information content of the community metagenome in order to characterise more specifically the community effort to break down the different lignocellulosic fractions. For two termite colonies (E.neo_1 and S.hey_1), biological replicates were analysed to ensure the repeatability and thus the validity of the generated sequencing results (Additional file 1: Figure S2). In our study, the sequencing effort resulted in over 730 million raw reads that were reduced to nearly 500 million reads after quality trimming and rRNA removal. Co-assembly of all generated metatranscriptomes resulted in 1,959,528 contigs, which were further taxonomically and functionally annotated using public databases. Additional details related to the metatranscriptomic analysis are summarised in Additional file 2: Table S1. Archaeal sequences were not very prevalent, and they accounted for less than 2% of the metatranscriptomic abundance, which somehow remains in agreement with another study on microbial metatranscriptomes in termite gut [10]. However, in contrast to the same study, little taxonomic consistency (including at the phylum level) was found between the bacterial community structure (Fig. 1) and the taxonomic distribution of assigned prokaryotic gene transcripts (Fig. 2), even though the initial database dependent taxonomy was further improved by comparing transcripts to MAGs reconstructed from termite gut microbiomes [34]. In particular, large under-representation of *Fibrobacteres* and over-representation of *Firmicutes* were observed in the termite gut prokaryotic metatranscriptomes, especially within the plant fibre-feeding termite cluster. The possible taxonomic misclassification of certain gene transcripts might stem from different factors, including under-representation of bacterial sequences (*Fibrobacteres* in particular) of termite origin in public databases [42] and extensive horizontal gene transfer occurring in bacteria [43]. For this reason, the phylogenetic distribution of the metabolic functions and pathways will not be broadly discussed in this study, especially in the case of the plant fibre-feeding termites.

In the studied prokaryotic metatranscriptomes, on average, 64.2% ± 2.7 of gene transcripts were assigned to 4910 KEGG Ontology categories (KO), accounting for an average of 62.4% ± 2.74 reads abundance per sample. Out of the annotated KOs, 2686 were further assigned a metabolic function (following annotation to KEGG BRITE database), and based on the calculated rarefaction curves, we could assume that a significant part of the higher termite gut metabolic potential was uncovered in our study (Additional file 1: Figure S7). Clustering microbial communities based on their metatranscriptomic profiles revealed the presence of two main clusters (Fig. 2c), nearly exactly resembling the separation of samples based on the community structure analysis (Fig. 1), which pointed towards putative differences in termite-specific activities of gut microbes. Slightly higher number of KO categories assigned for soil (3137.3 ± 438.5) versus plant fibre-feeding termite clusters (2663.2 ± 243.4) might relate to the host-specific metabolic needs (e.g. broader range of food sources) and reflects the overall higher species diversity of soil feeders gut microbiomes (Fig. 1b). In total, 70% of shared KOs accounted for 99.5% ± 0.2 of all function-assigned gene transcripts. Consequently, the 1168 KOs exclusively assigned to soil feeders cluster represented roughly 0.4% ± 0.1 of their metatranscriptomic abundance. For plant fibre feeders, the 328 specific KOs were even less abundant (0.2% ± 0.1).

We further compared the expression patterns of functionally assigned genes that were shared by the two clusters, and we found a surprisingly high congruency between soil and plant fibre feeder gut metatranscriptomes. We observed a significant correlation between the average cumulative expression of transcripts assigned to the same KO category for soil- and plant fibre-feeding termites (Fig. 2a), including the KOs assigned a metabolic function and in particular, for multiple glycosylases that were detected (Fig. 2b). The latter represented some of the most highly expressed KOs, confirming the specialization of the prokaryotic community towards carbohydrate metabolism. Further, metabolic pathway re-construction mirrored the above observations of functional congruency (Additional file 1: Figure S8). It also showed that cell motility (16.5% ± 0.7 and 12.8% ± 0.7 of metatranscriptomic abundance in soil- and plant fibre feeders clusters, respectively) together with carbohydrate metabolism (13.9% ± 2.0 and 18.7% ± 1.4 of metatranscriptomic abundance) were the two most highly expressed categories of metabolic pathways in both clusters (Fig. 2e). Finally, the relative abundance of different metabolic modules assigned to the carbohydrate metabolism category (Fig. 2f) was also similar among all the samples, regardless of the different nature and composition of plant fibre diet versus more decayed and nutrient rich soil, humus and litter.

Interestingly, K02406 (*fli*C gene) encoding for flagellin was by far the most expressed KO in both termite clusters. In total, transcripts involved in bacterial chemotaxis (ko02030) and flagellar assembly (ko02040) pathways accounted for 16.8% ± 4.5 and 10.4% ± 2.5 of all prokaryotic transcripts in plant fibre- and soil-feeding termite gut metatranscriptomes, respectively. According to the current taxonomic assignment, both *Spirochaetes* (56.3% ± 8.5 of transcripts assigned to this gene category) and *Firmicutes* (32.2% ± 5.9) were characterised with increased motility in soil feeders, while in plant fibre feeders, mainly *Spirochaetes* accounted for 81.2% ± 12.8 of bacteria actively swimming in the termite gut. The high expression of genes relevant to motility in these phyla could favour their abundance in the highly viscous environment of the termite gut. The tendency in our study remains consistent with [10] in which the wood-feeding *Nasutitermes corniger* gut microbiome was characterised with higher abundance of cell motility and chemotaxis assigned gene transcripts in comparison to the dung-feeding *Amitermes wheeleri*. In general, overrepresentation of transcripts relevant to cell motility in the termite gut versus other biomass-degrading microbiomes could be regarded as advantageous, enabling these prokaryotes, e.g. to actively reach their preferred substrates or to locate themselves in most favourable physicochemical gradients present within gut niches of their residence [10]. For comparison, in a previously characterised rumen metatranscriptome, genes involved in flagella assembly and chemotaxis were only poorly represented [44]. According to another study [45], genes related to cell motility and chemotaxis often co-cluster with CAZymes in bacterial genomes and show similar tendency of their expression profiles. That is why, next to the diverse CAZymes repertoire (discussed below), high bacterial cell mobility might to some extend contribute to the incredible success of the higher termite gut system in efficient biomass decomposition, often exceeding that of other lignocellulose utilising environments [4].

### Dietary imprints highlight subtle differences between the plant fibre- and soil-feeding termite gut symbiotic communities

Even though there was a strong conservation between the plant fibre- and soil-feeding termite clusters at different functional gene levels, to further investigate any possible cluster-specific functionalities, we used the linear discriminant analysis (LDA) size effect (LEfSe) [46] to determine if any metabolic trait could be putatively enriched in soil versus plant fibre feeders metatranscriptomes. General observations were similar to the previously published report related to the metagenomic and metatranscriptomic analysis of hindgut microbiota of wood- and dung-feeding higher termites [10]; therefore, they will be only briefly discussed here. Details are available in Additional file 2: Table S3. Next to the KOs exclusively assigned (though not necessarily abundant) to soil- and plant-fibre feeders, 56 and 174 different gene categories were significantly overrepresented in the plant fibre- and soil-feeding termite clusters, respectively. Illustration of the overrepresented KOs showed low metabolic overlap between the two clusters in terms of cluster-specific functionalities (Additional file 1: Figure S9). Several metabolic functions enriched in a plant fibre feeder cluster were related to nitrogen acquisition, with, e.g. atmospheric nitrogen fixation (e.g. *nif*D K02586 and *nif*K K02591—nitrogenase molybdenum-iron protein chains) being limited to this termite group. This observation is in line with previously published reports on wood-feeding termite microbiomes [9, 10]. In contrast to nitrogen-limited wood-based diet, soil has higher levels of fixed nitrogen in different forms, including nitrogenous residues of humic components derived from bacterial biomass. Therefore, multiple KOs relevant to protein degradation and amino acid metabolism (e.g. *psm*B K03433 proteasome β-subunit, *apr*X K17734 serine protease and *pep*E K05995 dipeptidase E) and bacterial cell wall degradation (e.g. *cwl*S K19220 peptidoglycan DL-endopeptidase, *lys*F/*cwl*E K19223 peptidoglycan DL-endopeptidase and *pda*A K01567 peptidoglycan-*N*-acetylmuramic acid deacetylase), as well as nitrate and nitrite transport and metabolism (e.g. *nar*G/*nar*Z/*nxr*A K00370 nitrate reductase/nitrite oxidoreductase α-subunit, *nir*S K15864 nitrite reductase and *nrt*D/*cyn*D K15579 nitrate/nitrite transport system ATP-binding protein) were enriched in soil-feeding termite gut metatranscriptomes.

In relation to sugar transport and metabolism, diverse components of sugar ATP-binding cassette (ABC) transporters were differentially enriched in both feeding categories; however. phosphotransferase system (PTS)-mediated sugar transport was largely enriched in a soil feeder cluster, including glucose, maltose, trehalose, *N*-acetylmuramic acid, fructose, mannitol and gluco- and galactosamine (Additional file 2: Table S3). Based on the enrichment of sugar isomerases, bacteria in the guts of plant fibre-feeding termites next to glucose would preferentially use xylose and arabinose for their metabolism (both derived from heteroxylans abundantly present in their woody diet). While their soil-feeding prokaryotic counterparts would rely mainly on glucose (e.g. cellulose and xyloglucans), ribose and galactosamine utilisation, the latter is present in the bacterial cell wall.

Interestingly, enrichment of soil-feeding termite cluster metatranscriptomes with CRISPR-Cas system-related components would indicate that bacteria in the termite gut actively use their adaptive immune system to protect themselves from invasive mobile genetic elements [47]. By briefly analysing spacer sequences from re-constructed CRISPRs and blasting them against NCBI viral database, we could potentially identify infecting phages (Additional file 2: Table S4). Most of the sequences corresponded to *Siphoviridae* and *Myoviridae* families of the order *Caudovirales*, which is in line with the dominance of these two types of dsDNA phages in the metavirome of the *Coptotermes formosanus* termite gut [48]. Homologous sequences to a few spacers were identified within the sequenced genomes of giant viruses, including the Pandoravirus with the largest known viral genome to date [49]. According to the very recent study, higher prevalence of huge phage in the human and animal gut compared to other environments is related to their main hosts which are *Firmicutes* and *Proteobacteria* [50], both phyla being more abundant in the soil-feeding termite cluster.

### Landscape of prokaryotic CAZymes in guts of plant fibre- and soil-feeding termites

**Fig. 3:**
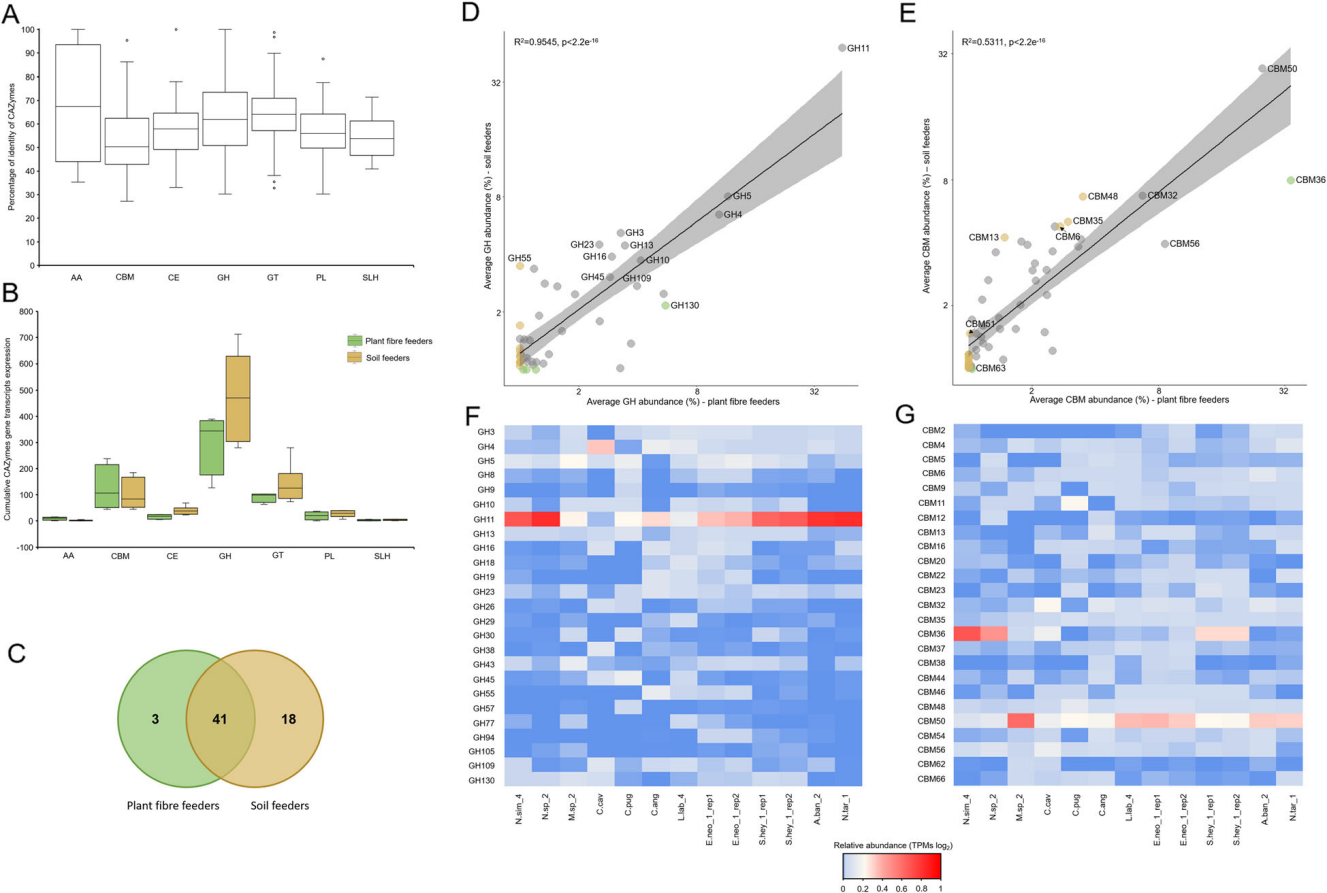
Prokaryotic CAZymes in the guts of plant fibre- and soil-feeding termites**. a** Box plot representation of the percentage identity of AAs, CBMs, CEs, GHs, GTs, PLs and SLHs in our dataset to the proteins in the NCBI non redundant protein database. **b** Cumulative expression of gene transcripts annotated to different CAZymes classes across plant fibre- and soil-feeding termites prokaryotic microbiomes. **c** Venn diagram representation of GH families common and exclusive to plant fibre- and soil-feeding termite clusters. **d** Average cumulative GH expression in gut prokaryotic microbiomes of plant fibre- and soil-feeding termites (dbCAN threshold of *e* value < 10^−18^ and coverage > 0.35); GHs enriched (LEfSE analysis) or present exclusively in plant fibre or soil feeder cluster are marked in green and brown colour, respectively. **e** Average cumulative CBM expression in gut prokaryotic microbiomes of plant fibre- and soil-feeding termites. CBMs enriched (LEfSE analysis) or present exclusively in plant fibre or soil feeder cluster are marked in green and brown colour, respectively. **f** Heatmap representation of the relative expression of major GH families across all prokaryotic microbiomes (dbCAN threshold of *e* value < 10^−18^ and coverage > 0.35). **g** Heatmap representation of the relative expression of major CBM families across all prokaryotic microbiomes

Prokaryotic contribution in terms of CAZymes expression is crucial for the functioning of the whole termite gut system [6], enabling the termite to feed on lignocellulose-rich biomass, which is particularly abundant in its diet. Until now, more attention has been given to CAZymes in wood-feeding higher termites [9–11, 15], whereas the termite genera which evolved to forage on more humified lignocellulose sources, including soil, humus or litter, have remained largely understudied. Initial analysis of metabolic pathways in our re-constructed de novo metatranscriptomes already evidenced the specialization of prokaryotic communities towards carbohydrate metabolism (Fig. 2e, f). To continue, all prokaryotic transcripts were compared to the entries in the CAZy database [12] and in total, 8920 putative CAZymes-related gene transcripts of prokaryotic origin were detected in our dataset. Generally, low sequence similarity of re-constructed CAZymes to carbohydrate active entries in the NCBI non redundant protein database (Fig. 3a) would indicate that the termite gut environment is a promising source of novel carbohydrate active enzymes. For this reason, and to avoid removing too many true positive CAZymes, the commonly applied threshold of *e* value < 10^−18^ and coverage > 0.35 when using the dbCAN tool for CAZymes discovery was not applied to our dataset, unless indicated otherwise.

On average, the discovered CAZymes accounted for 1.5% ± 0.3 of expressed prokaryotic gene transcripts in the different samples. In total, 8972 CAZyme domains were identified on the re-constructed CAZyme gene transcripts and assigned to 62 unique GHs families (3083 domains), 58 CBMs (2299 domains), 15 PL (159 domains), 12 CE (460 domains) and four families representing AA group (83 domains). GH was the most highly expressed CAZymes class across all the samples (Fig. 3b). Importantly, the recovered GH and CBM expression profiles for the replicates of colonies E.neo_1 and S.hey_1 showed high consistency, which suggest rather unbiased recovery of metabolic profiles when using the applied pipeline (Fig. 3). A large number of glycosyl transferases (GT; 27 families, 1333 domains) was detected; however, they were not further discussed here due to their biosynthetic nature of activities [12], which is of less interest to our study. Additionally, 84 cohesin/dockerin and 1472 SLH domains were also identified. As expected, due to the higher abundance of *Firmicutes* in the soil termite cluster, the average transcript abundance of cohesin/dockerin and SLH annotated genes was much higher in soil feeders than in plant fibre feeders. It would indicate the active presence of cellulosomes in the former group [51]. Gene transcript taxonomic assignment indicated that enzymes involved in carbohydrate metabolism originated mainly from *Firmicutes*, *Spirochaetes*, *Fibrobacteres* and *Bacteroidetes* (Additional file 1: Figure S10). While these results remain in agreement with [10], where many GH genes were predicted to be of Firmicutes origin, such high abundance of *Firmicutes*-related CAZymes seems questionable. Especially in the light of a previous study [9], where following the metagenomic binning, the majority of encoded GHs, initially assigned of *Firmicutes* origin, were re-assigned to either *Treponema* (*Spirochaetes*) or to *Fibrobacteres*. Therefore, the current taxonomic classification of CAZymes identified in this study could be further revised once additional prokaryotic genomes reconstructed from the termite gut microbiota are available.

Partially re-constructed transcripts containing more than one CAZymes ORF were detected in the de novo metatranscriptomic assembly, thus confirming a presence of putative saccharolytic operons in the termite gut microbes. Indeed, of the 8901 CAZymes-containing metatranscriptomic contigs, 276 were shown to contain at least two gene transcripts. Their abundance was similar in the two termite clusters (on average 3.4% ± 1.3 of reads) showing no prevalence of carbohydrate utilization gene clusters in a specific termite group. There was no evidence of the expression of polysaccharide utilization loci (PULs) typically found in *Bacteroidetes* genomes [52], in part due to high fragmentation of the metatranscriptome re-construction. However, higher expression of *susC* (*TonB*-dependent transporter) and *susD* (cell surface glycan-binding protein) genes, which together form part of PULs, coincided with higher abundance of *Bacteroidetes* in *C. cavifrons* guts. The above results remain in line with the recent functional metagenomics study of wood-feeding *Globitermes brachycerastes* gut microbiome, which revealed the tendency of saccharolytic genes to aggregate or form putative operons [11].

### Metabolic overlap and cluster specificities of carbohydrate-degrading strategies employed by microbes in plant fibre- and soil-feeding termite guts

Out of the 62 assigned GH families for the whole community metatranscriptome, 41 GHs were common, while 3 and 18 GHs were specific to plant fibre- or soil-feeding termite gut microbes, respectively (Fig. 3c). For the GH families that were expressed by the two community clusters, we observed a significant correlation of average gene expression levels (Fig. 3d). Still, even though the GH family diversity was comparable for soil- and plant fibre-feeding termite clusters, the number of different genes assigned to the same GH family was on average 1.5 times higher in soil-feeding termite gut microbiomes.

The CAZymes metatranscriptome of both feeding termite groups was dominated by gene transcripts assigned to the GH11 family (Fig. 3d, f, Additional file 2: Table S5), members of which have been predicted to have an endo-β-1,4-xylanase activity (EC 3.2.1.8). Transcriptional abundance of GH11 family is consistent with the recent study of a fibre-associated wood-feeding higher termite gut microbiome [15]. A high number of gene transcripts was also assigned to the GH5 family (mainly represented by GH5_4), which, based on their peptide pattern homology to characterised CAZyme proteins, were predicted to show either endoglucanase (EC 3.2.1.4) or endoxylanase activities (see below). Yet, the cumulative abundance of gene transcripts assigned to GH5 was lower than in the case of GH11. Another GH family, which initially appeared to be abundant (especially in microepiphytes feeding *C. cavifrons*), was GH109 (Additional file 1: Figure S11). The only so far identified activity of enzymes assigned to GH109 family is an α-*N*-acetylgalactosaminidase. These enzymes might be potentially involved in bacterial biomass turnover, by targeting the common components of bacterial cell walls, more specifically n-acetyl-d-galactosamine found in lipopolysaccharides [53]. However, following the application of the dbCAN threshold, its transcriptional abundance was significantly reduced (Fig. 3d), which better corresponded to the previously published reports. Still, active biomass turnover in animal guts was suggested to promote the release of biomass degrading enzymes from lysed bacterial cells to the gut lumen that subsequently become “public goods” helping other bacteria in lignocellulose degradation [54]. In this context, increased GH109 transcriptional expression may be indicatory of intense bacterial cell lysis in the termite gut. High transcriptional expression was also observed for other GH families, including GH4 with assigned maltose-6-phosphate glucosidase (EC 3.2.1.122) and α-galactosidase (EC 3.2.1.22) activities and GH23 putatively targeting bacterial peptidoglycan (Fig. 3d, f), and the latter again pointing to high bacterial biomass degradation rate. Although not excessively discussed in this study, chitin utilisation by the termite gut microbes seems high, based on the occurrence of putative β-*N*-acetylglucosaminidase/β-*N*-acetylhexosaminidase (EC 3.2.1.52), chitosanase (EC 3.2.1.132), α-1,3/1,4-L-fucosidase (EC 3.2.1.111), chitinase (EC 3.2.1.14) or chitin deacetylase (3.5.1.41), assigned to the GH3, GH8, GH29 and CE4 families as well as on the abundance of a CBM50 (putatively targeting chitin or peptidoglycan; Fig. 3e, g). Microbes in all studied termite guts were also able to preferentially utilise α-glycans, as it was assumed from high expression levels of GH13 assigned gene transcripts.

Based on the LEfSe analysis, three GH families (GH53, GH76 and GH130) were enriched in plant fibre-feeding termite metatranscriptomes, while GH55, GH65 and GH94 were more represented in a soil feeder cluster (Fig. 3d). In the case of a plant fibre feeder cluster, they presumably encoded endo-β-1,4-galactanase, α-1,6-mannanase, α-glucosidase or β-1,2-oligomannan phosphorylase activities. In contrast, families enriched in soil-feeding termites were mainly assigned as putative exo/endo-β-1,3-glucanase or cellobiose phosphorylase, trehalose, maltose phosphorylase or cellodextrin phosphorylase. In line with increased utilisation of the two major lignocellulose components (Fig. 3d, f), meaning cellulose and xylan, respective xylan-targeting CBM36 was slightly enriched in a plant fibre cluster, while cellulose-specific CBM6 was more abundant in soil-feeding termite gut metatranscriptome (Fig. 3e, g). Interestingly, glycogen-binding domain CBM48 was enriched in soil feeders (Fig. 3e, g), and together with the enrichment of the glycogen phosphorylase coding genes (K00688, Additional file 2: Table S3), it would indicate intensive glycogen utilisation by the termite gut bacteria. In general, glycogen is a major intracellular reserve polymer of yeast and bacteria [55]; therefore, it might be also abundantly present in soil microbial biomass, which is the main diet component of soil-feeding higher termites.

The diversity and gene expression profiles of microbial CAZymes identified in our study remain in agreement with previous metatranscriptomic reports published for wood-feeding termites [10, 15]. Very little information is available on humus, soil and litter feeders, except for one previous metagenomic analysis of microbiota in the hindguts of six different wood- and soil-feeding higher termites [42]. Yet, extrapolation of the metagenomic results to functional gene expression profiles revealed by metatranscriptomics is difficult to achieve and thus the two studies cannot be directly compared. Previously, reported metatranscriptome of the dung-feeding termite, *A. wheeleri,* is the closest study that could be compared with our soil cluster [10]. Accordingly, transcriptomic abundance of mainly GH11, GH5, GH3 and GH10 families was consistent with our results.

### Specific degradation of the different lignocellulose fractions by the termite gut microbial enzymes

**Fig. 4:**
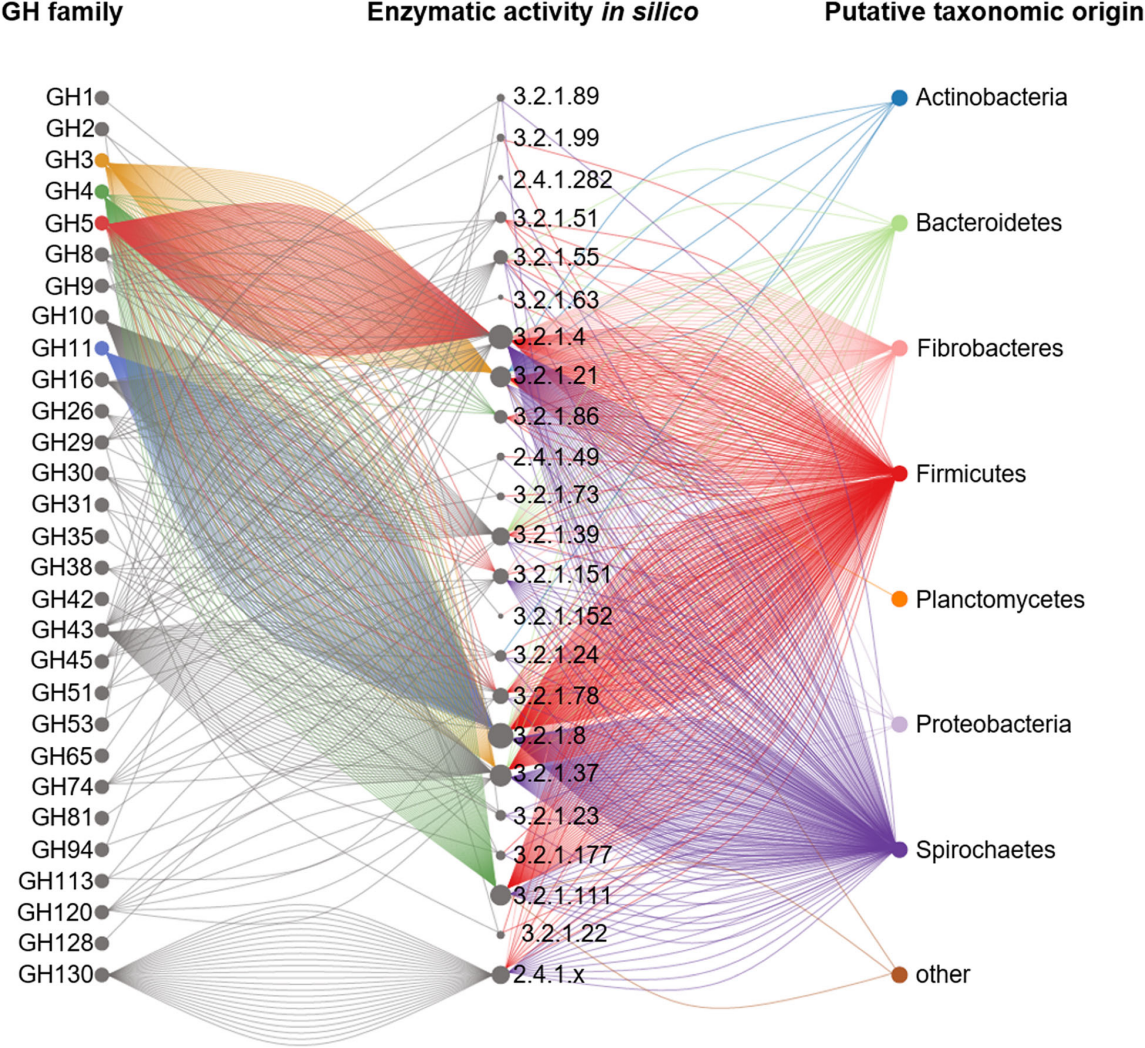
Metabolic activities assigned to different GH families following homology search to peptide pattern analysis (Hotpep). Only the most relevant EC classes are displayed. The size of the node specific to the EC corresponds to the number of the re-constructed genes transcripts. Some of the abundant glycoside hydrolase families are marked with colour to improve the visibility. Putative taxonomic origin of re-constructed gene transcripts annotated to EC classes was assigned with IMG MER and comparison of gene transcripts to reconstructed MAGs of termite origin [34]

**Fig. 5:**
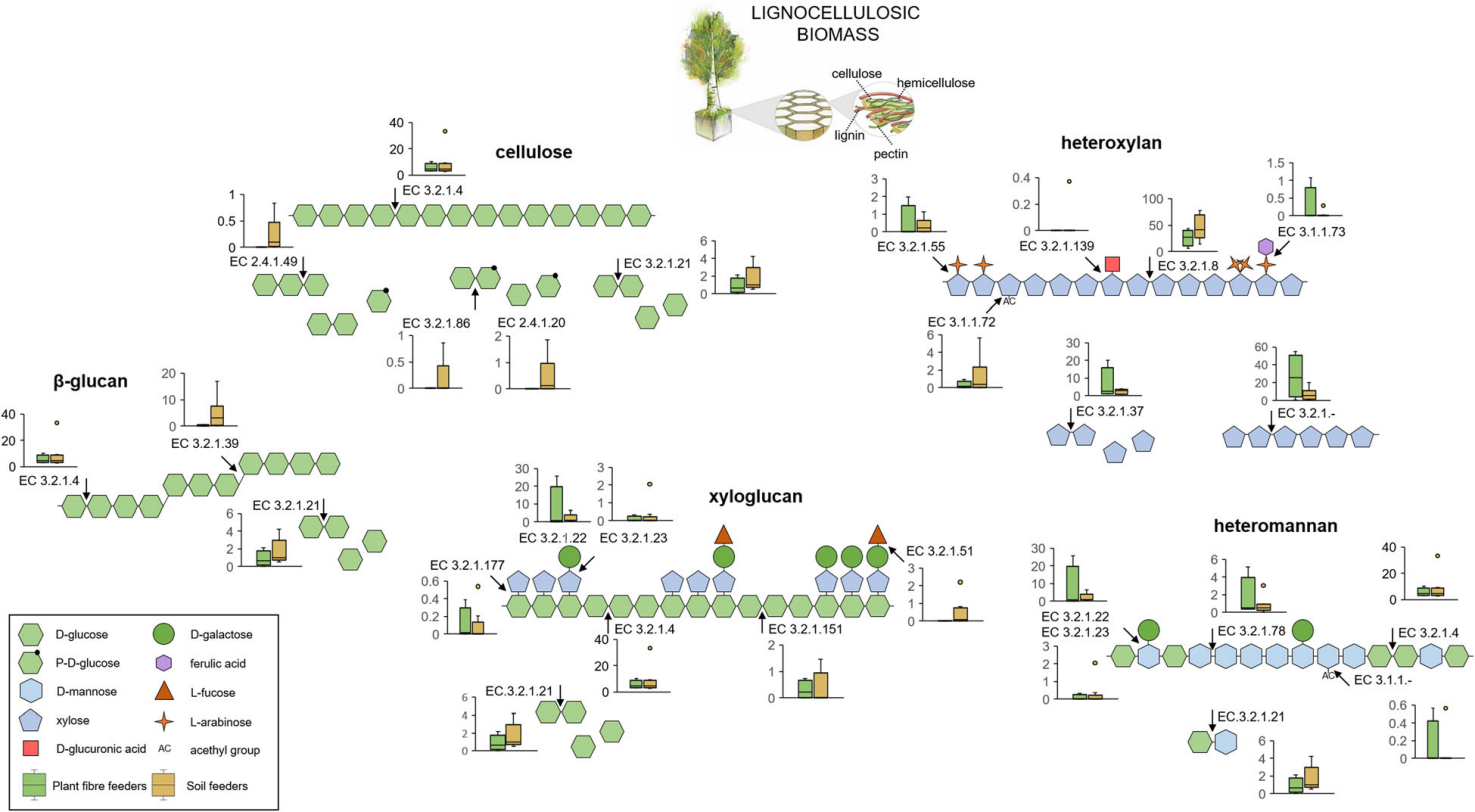
Schematic representation of the enzymatic decomposition of different lignocellulose components. Most relevant enzymatic functionalities identified based on the abundance of gene transcripts assigned to EC classes are presented for plant fibre- and soil-feeding termite clusters. The y-axis of the box plots represents the percentage of all hydrolytic gene transcripts

It is well recognised that multiple enzymatic activities can be assigned to a single CAZyme family, e.g. including GH3, GH5 and GH13. Therefore, to get more insights into the putative enzymatic activities, gene transcripts encoding for CAZymes in our study were further given an EC number using homology search to peptide pattern (Hotpep) [36]. Previously, the study of carbohydrate hydrolytic potential in anaerobic digester showed the usefulness of the complementary Hotpep analysis to the dbCAN-mediated CAZymes-coding discovery [56]. In that study, several in silico predicted enzymatic activities were further experimentally confirmed. Here, 921 prokaryotic gene transcripts were given EC numbers. Their assignment indeed showed that gene transcripts classified to, e.g. GH5 family were further given different enzymatic activities in silico (mainly 3.2.1.4, followed by 3.2.1.x, 3.2.1.151 and 3.2.1.78, Fig. 4). According to the predicted enzymatic activities, the most highly abundant enzymes were the ones targeting the backbone of the different lignocellulose components (Fig. 5), including mainly endocellulases (cellulose and presumably xylo- and β-glucans; EC 3.2.1.4) and endoxylanases (heteroxylans; EC 3.2.1.8) and to a lesser extent endomannanases (heteromannan, EC 3.2.1.78). Most of the respective gene transcripts were taxonomically assigned to *Firmicutes*, *Fibrobacteres* and *Spirochaetes*; however, due to largely incomplete public databases and small number of available MAGs of termite origin, this taxonomic classification should be revised when more representatives of the termite gut microbiome have their genomes sequenced. Complete utilisation of cellulose and xylan by the two termite clusters gut microbiomes could be further confirmed by abundant expression of genes encoding for β-glucosidases (EC 3.2.1.21, EC 3.2.1.86) and xylosidases (EC 3.2.1.37), as well as the presence of respective sugar transporters and isomerases (discussed above). Concerning mannan utilisation, no putative mannosidase (EC 3.2.1.25) was detected in our dataset. However, as previously shown for other anaerobic biomass degrading environments, a combined action of *N*-Acyl-d-glucosamine 2-epimerase and 4-*O*-β-D-mannosyl-d-glucose phosphorylase would first transform mannobiose into β-d-mannosyl-(1 → 4)-d-glucose, with the subsequent hydrolysis of mannosylglucose to glucose and mannose-1-phosphate [56]. Both enzymatic categories were characterised with relatively high gene expression levels in fibre- and soil-feeding termite datasets, pointing to a similar mechanism of mannobiose hydrolysis in the termite gut.

Next to cellulose and lignin, mannans and xylans (hemicellulose) are the main components of woody biomass [3], and both polymers can be largely substituted with, e.g. arabinose, galactose, xylose, glucuronic acid and other simple sugar monomers. Accordingly, in the case of plant fibre-feeding termites, multiple debranching enzymes, including α-arabinofuranosidases (EC 3.2.1.55), α-galactosidases (EC 3.2.1.22, EC 3.2.1.23) and α-xylosidases (EC 3.2.1.177), were slightly more abundant. Ferulic acid is the most abundant hydroxycinnamic acid in the plant cell wall, and it is ester-linked to the cell wall polysaccharide arabinoxylan [57]. By forming covalent linkages between polysaccharide chains (cross-linking) and polysaccharide and lignin components, it limits the accessibility and thus the digestibility of polysaccharides in plant biomass. Consequently, its higher expression was identified in plant fibre feeders; however, the discovery rate of putative feruloyl esterases (EC 3.1.1.73) was low in our study and mainly limited to the metatranscriptome of the wood-feeding *Microcerotermes* (M.sp_2). Nevertheless, expression of trans-feruloyl-CoA hydratase/vanillin synthase (K18383) genes in several wood- and soil-feeding termite gut microbiomes indicates that bacteria can further metabolise ferulic acid to vanillin, as a part of their secondary metabolism. By contrast, average cumulative expression of putative acetyl esterases (EC 3.1.1.72) was higher in soil feeders.

### Community-wide lignocellulolytic phenotype of different termite gut microbiomes is contributed by distinct multiple and single bacterial players

Direct comparison of the re-constructed gene transcripts showed that roughly 0.18% of all de novo re-constructed prokaryotic genes were shared between all the samples. These values were slightly higher inside the two clusters, and 0.49% of transcripts were shared between the samples assigned to plant fibre feeders. For soil feeders, slightly less of the total repertoire of expressed genes was common to all of the studied gut microbiomes (most probably due to higher species diversity), with roughly 0.29% of shared transcripts. Yet, gene transcript assignment to broader functional categories and subsequent enrichment of common KOs in gut metatranscriptomes of both types of termite feeding diets provides an example of the functional congruency. This observation indicates that even though each termite species operates with its own gut bacteria, there is a functional equivalence in microbial populations across different termite hosts. Taxonomically, distinct microbial communities, displaying conserved global functional profiles, have been previously reported in other environments, including anoxic waste water treatment tanks [58] and marine sponges [59]. Moreover, in the previous metagenomic and metatranscriptomic study of two higher termite species, the convergence of functions essential to termite biology among the gut microbiomes of wood and dung feeders has already been proposed [10].

Based on the number of different genes assigned to the same functional category, the diversity of microbes contributing to the observed phenotype was 1.65 ± 1.2 fold higher in gut microbiomes of soil-feeding termites versus their plant fibre-feeding counterparts. It makes a direct link with the significantly higher bacterial compositional diversity in soil versus plant fibre-feeding termites, as discussed above (Fig. 1b). For most of the assigned functionalities, we could also observe a significant correlation between the number of gene transcripts assigned to a gene category and its cumulative expression per sample (Additional file 1: Figure S12). It would suggest that most of the observed microbial processes are the collective metabolisms of multiple taxa contributing to a particular ecosystem trait, rather than the metabolic dominance of single prokaryotic players.

**Fig. 6:**
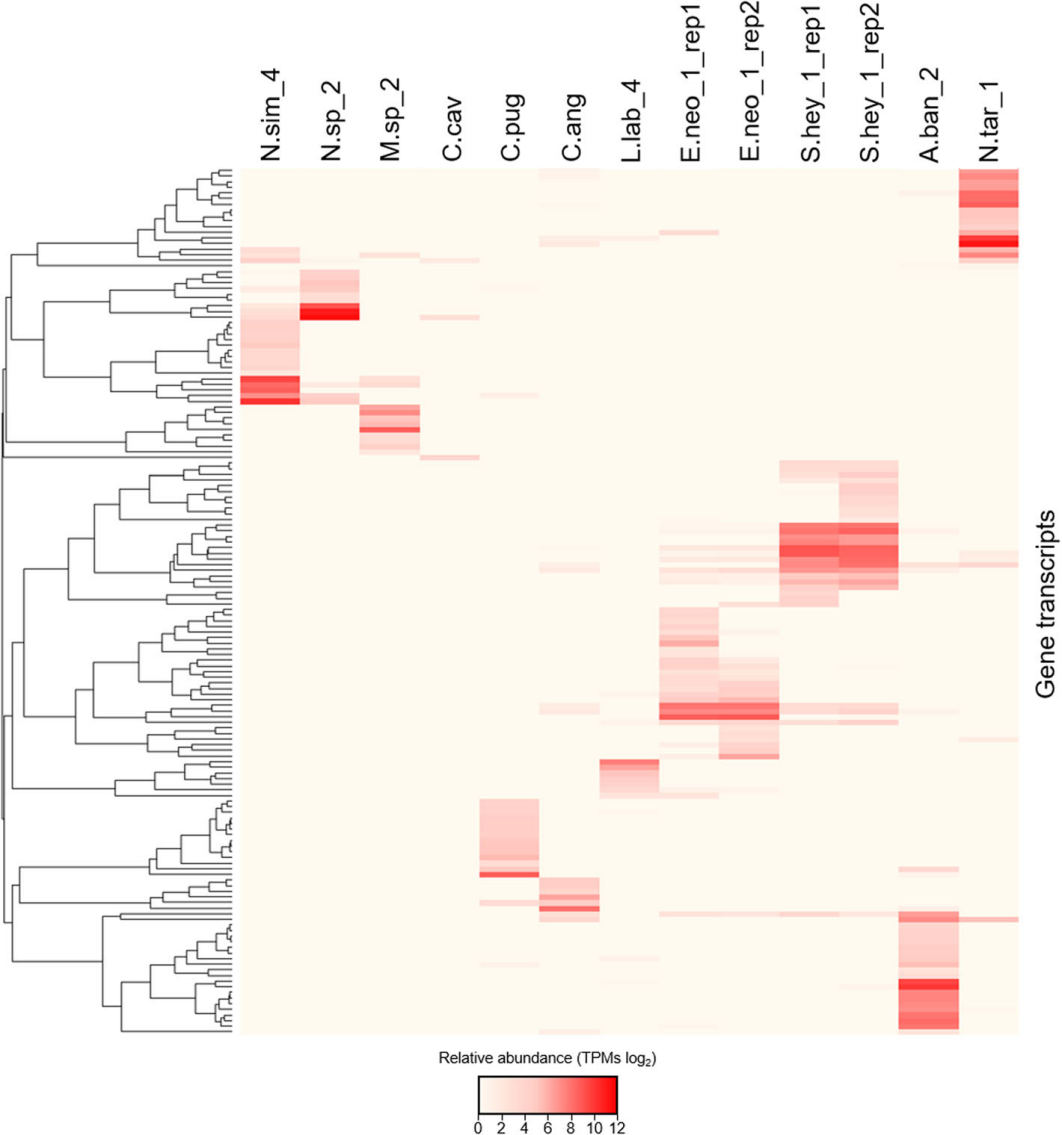
GH11 expression profiles of all the samples**.** Clustering is based on relative abundance (log_2_) of all gene transcripts assigned to GH11 family. Each row on a heatmap represents one gene

Functional gene redundancy implies that similar metabolic functions are expressed by multiple bacteria [60]. In the case of the termite gut, it can be directly extrapolated to upper levels of functional hierarchy including CAZyme diversity profiles and even their gene expression patterns. For example, the occurrence and abundance of different gene transcripts assigned to the GH11 family strongly differ between all the samples, pointing towards the unique gene repertoire of each prokaryotic metatranscriptome (Fig. 6). It is also interesting to note that several GH11 gene transcripts were characterised with exceptionally high expression levels, compared to the average expression levels of the remaining GH11 assigned genes (Additional file 1: Figure S13). The attribution of over 80% of the sequencing reads to roughly 21.8% ± 9.5 of putative GH11 gene transcripts would indicate that the degradation of the xylan backbone is confined to single microbial players rather than to multiple microbial populations. Similar results, with few CAZyme outliers compared to the average expression levels of genes assigned to a given GH family were shown for, e.g. GH5 or GH10. Altogether, it indicates that next to the combined activity of multiple microbial populations, single bacterial players may also contribute to some of the observed lignocellulolytic phenotypes of the termite gut system. However, in none of the cases, the abundance of the most highly abundant transcripts was comparable to the GH11 family outliers. From the application point of view, such enzymes are potentially interesting candidates for further bioprospecting, once their factual activity is biochemically characterised.

## Conclusions

The host diet is recognized to be one of the major determinants of the bacterial community structure in higher termite guts. However, mainly termite species feeding on wood have been investigated so far. In this study, using integrative targeted metagenomics (16S rRNA gene amplicons) and metatranscriptomics (enriched prokaryotic mRNA), we analysed gut bacterial profiles of different higher termites, feeding on diverse substrates, including wood, grass, litter, humus, soil and epiphytes. Thus, we expanded the knowledge on lignocellulolytic capacities of gut bacteria from termites feeding on biomass other than wood. Our results clearly evidenced that regardless of the feeding habit, the prokaryotic communities are specialised in the direction of carbohydrates metabolism, and that they share a majority of their metabolic signatures. Still, following the dietary imprints, subtle differences were identified for plant fibre feeders and soil feeders. Importantly, our results showed that each termite species is a unique organism operating with its own bacterial flora and accompanying gene transcripts. Yet, there is a functional equivalence between microbial populations across different termite hosts. Although the chosen metatranscriptomic approach gave an excellent overview of the community effort to break down the different lignocellulosic components, the further metagenomic binning and reference-independent taxonomic classification of re-constructed microbial genomes would be beneficial to assign specific functions to bacterial lineages within the termite gut. It would also help reconstructing complete gene sequences necessary to proceed with characterisation of the most promising CAZy proteins. Moreover, the integrative metatranscriptomic and metagenomic analyses would be particularly useful if they were applied to both prokaryotic and termite samples originating from different segments of highly compartmented termite gut. It would allow for even better insight into the host and symbionts interplay across the different niches of this unique environment.

## Supplementary information

**Additional file 1: Figure S1.** 16S rRNA gene amplicon sequencing results for triplicates, presented for 10 selected samples. **Figure S2.** Duplicates (biological replicates) of metatranscriptomic libraries for two selected colonies (E.neo_1, S.hey_1). **Figure S3.** The observed richness estimator rarefaction curves based on high-throughput amplicon sequencing of 16S rRNA gene for 41 tested samples of termite gut bacteria. **Figure S4.** Tree based on the calculated Jaccard similarity in bacterial community membership, based on 16S rRNA gene amplicon sequencing. **Figure S5.** 3D-NMDS ordination of the calculated Bray-Curtis dissimilarity (A) and Jaccard similarity (B) in bacterial community structures at the OTU level. **Figure S6.** 3D-NMDS ordination of the weighted (A) and unweighted (B) UniFrac-calculated pairwise distance across all samples. **Figure S7.** The calculated rarefaction curves of all the captured enzymatic annotations (reflected by the assigned KEGG BTITE enzyme numbers). **Figure S8.** Average expression of pathways (cumulative abundance of transcripts assigned to given pathway) across prokaryotic microbiomes of plant fibre- and soil-feeding termites. **Figure S9.** Illustration of the overrepresented KEGG Ontology categories showing low metabolic overlap between the two clusters in terms of cluster-specific functionalities. **Figure S10.** Sequence homology-based taxonomic prediction of prokaryotic groups contributing the putative CAZymes expression in plant fibre- and soil-feeding termites. **Figure S11.** Average GH expression in prokaryotic gut microbiomes of plant fibre- and soil-feeding termites (results without application of the dbCAN tool threshold of e-value <10^−18^ and coverage >0.35). **Figure S12.** Correlation between the number of gene transcripts assigned to a gene category and its cumulative expression per sample. **Figure S13.** Expression of the gene transcripts assigned to GH11 CAZy family across all prokaryotic microbiomes.

**Additional file 2: Table S1.** Details of metatranscriptomic sequencing of 11 selected samples. **Table S2.** OTU table based on bacterial 16S rRNA gene amplicon sequencing for 41 tested higher termite guts samples, with taxonomic annotation based on DicDB database. **Table S3.** KO categories enriched or present exclusively in plant fibre- or soil-feeding termite cluster. **Table S4.** CRISPR-Cas system related components identified in the analysed metatranscriptomes. **Table S5.** The relative abundances of 25 top expressed GH families identified in this study.

## Data Availability

Metatranscriptomic sequencing results are available in the Sequence Read Archive (SRA) database under the accession number SRP135739. The 16S rRNA gene amplicon sequencing results are available in the SRA database under accession number SRP135739. Partial CO-II gene sequencing results are available in GenBank under accession numbers from MH067978 to MH068018.

## Abbreviations

AA: Auxiliary activity
ABC: ATP-binding cassette
CAZymes: Carbohydrate-active enzymes
CBM: Carbohydrate-binding module
CE: Carbohydrate esterase
CO-II: Cytochrome oxidase subunit 2
EC number: The Enzyme Commission number
GH: Glycoside hydrolase
KO: KEGG Ontology categories
ORF: Open reading frame
OTUs: Operational taxonomic units
PL: Polysaccharide lyase
PTS: Phosphotransferase system
PUL: Polysaccharide utilization loci
SRA: Sequence Read Archive
TPMs: Transcripts per million

## References

[1] Jouquet P, Traoré S, Choosai C, Hartmann C, Bignell D (2011). Influence of termites on ecosystem functioning. Ecosystem services provided by termites. Eur J Soil Biol.

[2] Donovan S, Eggleton P, Bignell D (2001). Gut content analysis and a new feeding group classification of termites. Ecol Entomol.

[3] Doblin MS, Pettolino F, Bacic A (2010). Plant cell walls: the skeleton of the plant world. Funct Plant Biol.

[4] Xie L, Liu N, Huang Y. Lignocellulose degradation in termite symbiotic systems, in biological conversion of biomass for fuels and chemicals: explorations from natural utilization systems. 2013. Royal Society of Chemistry.

[5] Watanabe H, Tokuda G (2010). Cellulolytic systems in insects. Annu Rev Entomol.

[6] Brune A (2014). Symbiotic digestion of lignocellulose in termite guts. Nat Rev Microbiol.

[7] Bourguignon T, Lo N, Dietrich C, Šobotník J, Sidek S, Roisin Y (2018). *Rampant host switching shaped the termite gut microbiome*. Current Biology.

[8] da Costa RR, Poulsen M (2018). Mixed-mode transmission shapes termite gut community assemblies. Trends Microbiol.

[9] Warnecke F, Luginbühl P, Ivanova N, Ghassemian M, Richardson TH, Stege JT (2007). Metagenomic and functional analysis of hindgut microbiota of a wood-feeding higher termite. Nature.

[10] He S, Ivanova N, Kirton E, Allgaier M, Bergin C, Scheffrahn RH (2013). Comparative metagenomic and metatranscriptomic analysis of hindgut paunch microbiota in wood-and dung-feeding higher termites. PLoS One.

[11] Liu N, Li H, Chevrette MG, Zhang L, Cao L, Zhou H (2019). Functional metagenomics reveals abundant polysaccharide-degrading gene clusters and cellobiose utilization pathways within gut microbiota of a wood-feeding higher termite. ISME J.

[12] Cantarel BL, Coutinho PM, Rancurel C, Bernard T, Lombard V, Henrissat B (2008). The Carbohydrate-Active EnZymes database (CAZy): an expert resource for glycogenomics. Nucleic Acids Res.

[13] Boraston AB, Bolam DN, Gilbert HJ, Davies GJ (2004). Carbohydrate-binding modules: fine-tuning polysaccharide recognition. Biochem J.

[14] Marynowska M, Goux X, Sillam-Dussès D, Rouland-Lefèvre C, Roisin Y, Delfosse P (2017). Optimization of a metatranscriptomic approach to study the lignocellulolytic potential of the higher termite gut microbiome. BMC Genomics.

[15] Tokuda G, Mikaelyan A, Fukui C, Matsuura Y, Watanabe H, Fujishima M (2018). Fiber-associated spirochetes are major agents of hemicellulose degradation in the hindgut of wood-feeding higher termites. Proc Natl Acad Sci.

[16] Jones DT, Eggleton P (2010). Global biogeography of termites: a compilation of sources, in Biology of termites: a modern synthesis.

[17] Bourguignon T, Šobotnik J, Lepoint G, Martin JM, Hardy OJ, Dejean A (2011). Feeding ecology and phylogenetic structure of a complex neotropical termite assemblage, revealed by nitrogen stable isotope ratios. Ecol Entomol.

[18] Cuezzo C, Carrijo TF, Cancello EM (2015). Transfer of two species from *Nasutitermes* Dudley to *Cortaritermes* Mathews (Isoptera: Termitidae: Nasutitermitinae). Austral Entomol.

[19] Miura T, Roisin Y, Matsumoto T (2000). *Molecular phylogeny and biogeography of the nasute termite genus* Nasutitermes *(Isoptera: Termitidae) in the Pacific tropics*. Mol Phylogenet Evol.

[20] Klindworth A, Pruesse E, Schweer T, Peplies J, Quast C, Horn M (2013). Evaluation of general 16S ribosomal RNA gene PCR primers for classical and next-generation sequencing-based diversity studies. Nucleic Acids Res.

[21] Edgar RC (2010). Search and clustering orders of magnitude faster than BLAST. Bioinformatics.

[22] Mikaelyan A, Köhler T, Lampert N, Rohland J, Boga H, Meuser K (2015). Classifying the bacterial gut microbiota of termites and cockroaches: a curated phylogenetic reference database (DictDb). Syst Appl Microbiol.

[23] Schloss PD, Westcott SL, Ryabin T, Hall JR, Hartmann M, Hollister EB (2009). Introducing mothur: open-source, platform-independent, community-supported software for describing and comparing microbial communities. Appl Environ Microbiol.

[24] Team RC (2013). R: a language and environment for statistical computing.

[25] Oksanen J, Blanchet F, Kindt R, Legendre P, O’Hara R, Vegan: community ecology package. R Package 2.3-3. R Foundation for statistical computing Vienna. Austria. 2016.

[26] Lozupone C, Lladser ME, Knights D, Stombaugh J, Knight R (2011). UniFrac: an effective distance metric for microbial community comparison. ISME J.

[27] Edgar RC (2004). MUSCLE: multiple sequence alignment with high accuracy and high throughput. Nucleic Acids Res.

[28] Gouveia-Oliveira R, Sackett PW, Pedersen AG (2007). MaxAlign: maximizing usable data in an alignment. BMC Bioinformatics.

[29] Price MN, Dehal PS, Arkin AP (2010). FastTree 2–approximately maximum-likelihood trees for large alignments. PLoS One.

[30] Bhagwat AA, Ying ZI, Smith A (2014). Evaluation of ribosomal RNA removal protocols for salmonella RNA-Seq projects. Adv Microbiol.

[31] Giannoukos G, Ciulla DM, Huang K, Haas BJ, Izard J, Levin JZ (2012). Efficient and robust RNA-seq process for cultured bacteria and complex community transcriptomes. Genome Biol.

[32] Kopylova E, Noé L, Touzet H (2012). SortMeRNA: fast and accurate filtering of ribosomal RNAs in metatranscriptomic data. Bioinformatics.

[33] Markowitz VM, Chen I-MA, Palaniappan K, Chu K, Szeto E, Grechkin Y (2011). IMG: the integrated microbial genomes database and comparative analysis system. Nucleic Acids Res.

[34] Hervé V, Liu P, Dietrich C, Sillam-Dussès D, Stiblik P, Šobotník J (2020). Phylogenomic analysis of 589 metagenome-assembled genomes encompassing all major prokaryotic lineages from the gut of higher termites. PeerJ.

[35] Yin Y, Mao X, Yang J, Chen X, Mao F, Xu Y (2012). *dbCAN: a web resource for automated carbohydrate-active enzyme annotation*. Nucleic Acids Res.

[36] Busk PK, Pilgaard B, Lezyk MJ, Meyer AS, Lange L (2017). Homology to peptide pattern for annotation of carbohydrate-active enzymes and prediction of function. BMC Bioinformatics.

[37] Li B, Ruotti V, Stewart RM, Thomson JA, Dewey CN (2010). RNA-Seq gene expression estimation with read mapping uncertainty. Bioinformatics.

[38] Mikaelyan A, Dietrich C, Köhler T, Poulsen M, Sillam-Dussès D, Brune A (2015). Diet is the primary determinant of bacterial community structure in the guts of higher termites. Mol Ecol.

[39] Benjamino J, Lincoln S, Srivastava R, Graf J (2018). Low-abundant bacteria drive compositional changes in the gut microbiota after dietary alteration. Microbiome.

[40] Rahman NA, Parks DH, Willner DL, Engelbrektson AL, Goffredi SK, Warnecke F (2015). A molecular survey of Australian and north American termite genera indicates that vertical inheritance is the primary force shaping termite gut microbiomes. Microbiome.

[41] Dietrich C, Köhler T, Brune A (2014). The cockroach origin of the termite gut microbiota: patterns in bacterial community structure reflect major evolutionary events. Appl Environ Microbiol.

[42] Rossmassler K, Dietrich C, Thompson C, Mikaelyan A, Nonoh JO, Scheffrahn RH (2015). Metagenomic analysis of the microbiota in the highly compartmented hindguts of six wood-or soil-feeding higher termites. Microbiome.

[43] Koonin EV, Makarova KS, Aravind L (2001). Horizontal gene transfer in prokaryotes: quantification and classification. Ann Rev Microbiol.

[44] Jiang Y, Xiong X, Danska J, Parkinson J (2016). Metatranscriptomic analysis of diverse microbial communities reveals core metabolic pathways and microbiome-specific functionality. Microbiome.

[45] Calusinska, M., M. Marynowska, M. Bertucci, B. Untereiner, D. Klimek, X. Goux, et al., Targeted biomass degradation by the higher termite gut system-integrative omics applied to host and its gut microbiome. bioRxiv, 2020.

[46] Segata N, Izard J, Waldron L, Gevers D, Miropolsky L, Garrett WS (2011). Metagenomic biomarker discovery and explanation. Genome Biol.

[47] Briner AE, Barrangou R (2016). Deciphering and shaping bacterial diversity through CRISPR. Curr Opin Microbiol.

[48] Tikhe CV, Husseneder C (2018). Metavirome sequencing of the termite gut reveals the presence of an unexplored bacteriophage community. Front Microbiol.

[49] Philippe N, Legendre M, Doutre G, Couté Y, Poirot O, Lescot M (2013). Pandoraviruses: amoeba viruses with genomes up to 2.5 Mb reaching that of parasitic eukaryotes. Science.

[50] Al-Shayeb B, Sachdeva R, Chen L-X, Ward F, Munk P, Devoto A, et al. Clades of huge phage from across Earth's ecosystems. BioRxiv. 2019;572362. 10.1038/s41586-020-2007-4 PMC7162821 32051592

[51] Terry SA, Badhan A, Wang Y, Chaves AV, McAllister TA (2019). Fibre digestion by rumen microbiota–a review of recent metagenomic and metatranscriptomic studies. Can J Anim Sci.

[52] Grondin JM, Tamura K, Déjean G, Abbott DW, Brumer H (2017). Polysaccharide utilization loci: fueling microbial communities. J Bacteriol.

[53] Leyn, S.A., F. Gao, C. Yang, and D.A. Rodionov, N-Acetylgalactosamine utilizationpathway and regulon in proteobacteria genomic and experimental characterization in Shewanella*.* J Biol Chem, 2012. **287**(33): p. 28047-28056. 10.1074/jbc.M112.382333 PMC3431674 22711537

[54] Feng G, Flanagan BM, Mikkelsen D, Williams BA, Yu W, Gilbert RG (2018). Mechanisms of utilisation of arabinoxylans by a porcine faecal inoculum: competition and co-operation. Sci Rep.

[55] Wilson WA, Roach PJ, Montero M, Baroja-Fernández E, Muñoz FJ, Eydallin G (2010). Regulation of glycogen metabolism in yeast and bacteria. FEMS Microbiol Rev.

[56] Bertucci M, Calusinska M, Goux X, Rouland-Lefèvre C, Untereiner B, Ferrer P (2019). Carbohydrate hydrolytic potential and redundancy of anaerobic digestion microbiome exposed to acidosis uncovered by metagenomics. Appl Environ Microbiol.

[57] Mathew S, Abraham TE (2004). Ferulic acid: an antioxidant found naturally in plant cell walls and feruloyl esterases involved in its release and their applications. Crit Rev Biotechnol.

[58] Roume H, Heintz-Buschart A, Muller EE, May P, Satagopam VP, Laczny CC (2015). Comparative integrated omics: identification of key functionalities in microbial community-wide metabolic networks. NPJ Biofilms Microbiomes.

[59] Fan L, Reynolds D, Liu M, Stark M, Kjelleberg S, Webster NS (2012). Functional equivalence and evolutionary convergence in complex communities of microbial sponge symbionts. Proc Natl Acad Sci.

[60] Heintz-Buschart A, Wilmes P (2018). Human gut microbiome: function matters. Trends Microbiol.

