# Supplementary material for: Compositional and functional characterisation of biomass-degrading microbial communities in guts of plant fibre- and soil-feeding higher termites
Source: Microbiome. 2020 Jun 23;8:96. doi: 10.1186/s40168-020-00872-3 (PMC7313118; doi:10.1186/s40168-020-00872-3)
Supplement: Supplementary file 2 — Additional file 1: Figure S1. 16S rRNA gene amplicon sequencing results for triplicates, presented for 10 selected samples. Figure S2. Duplicates (biological replicates) of metatranscriptomic libraries for two selected colonies (E.neo_1, S.hey_1). Figure S3. The observed richness estimator rarefaction curves based on high-throughput amplicon sequencing of 16S rRNA gene for 41 tested samples of termite gut bacteria. Figure S4. Tree based on the calculated Jaccard similarity in bacterial community membership, based on 16S rRNA gene amplicon sequencing. Figure S5. 3D-NMDS ordination of the calculated Bray-Curtis dissimilarity (A) and Jaccard similarity (B) in bacterial community structures at the OTU level. Figure S6. 3D-NMDS ordination of the weighted (A) and unweighted (B) UniFrac-calculated pairwise distance across all samples. Figure S7. The calculated rarefaction curves of all the captured enzymatic annotations (reflected by the assigned KEGG BTITE enzyme numbers). Figure S8. Average expression of pathways (cumulative abundance of transcripts assigned to given pathway) across prokaryotic microbiomes of plant fibre- and soil-feeding termites. Figure S9. Illustration of the overrepresented KEGG Ontology categories showing low metabolic overlap between the two clusters in terms of cluster-specific functionalities. Figure S10. Sequence homology-based taxonomic prediction of prokaryotic groups contributing the putative CAZymes expression in plant fibre- and soil-feeding termites. Figure S11. Average GH expression in prokaryotic gut microbiomes of plant fibre- and soil-feeding termites (results without application of the dbCAN tool threshold of e-value <10−18 and coverage >0.35). Figure S12. Correlation between the number of gene transcripts assigned to a gene category and its cumulative expression per sample. Figure S13. Expression of the gene transcripts assigned to GH11 CAZy family across all prokaryotic microbiomes. [file 40168_2020_872_MOESM1_ESM.docx]

**Additional file 1**


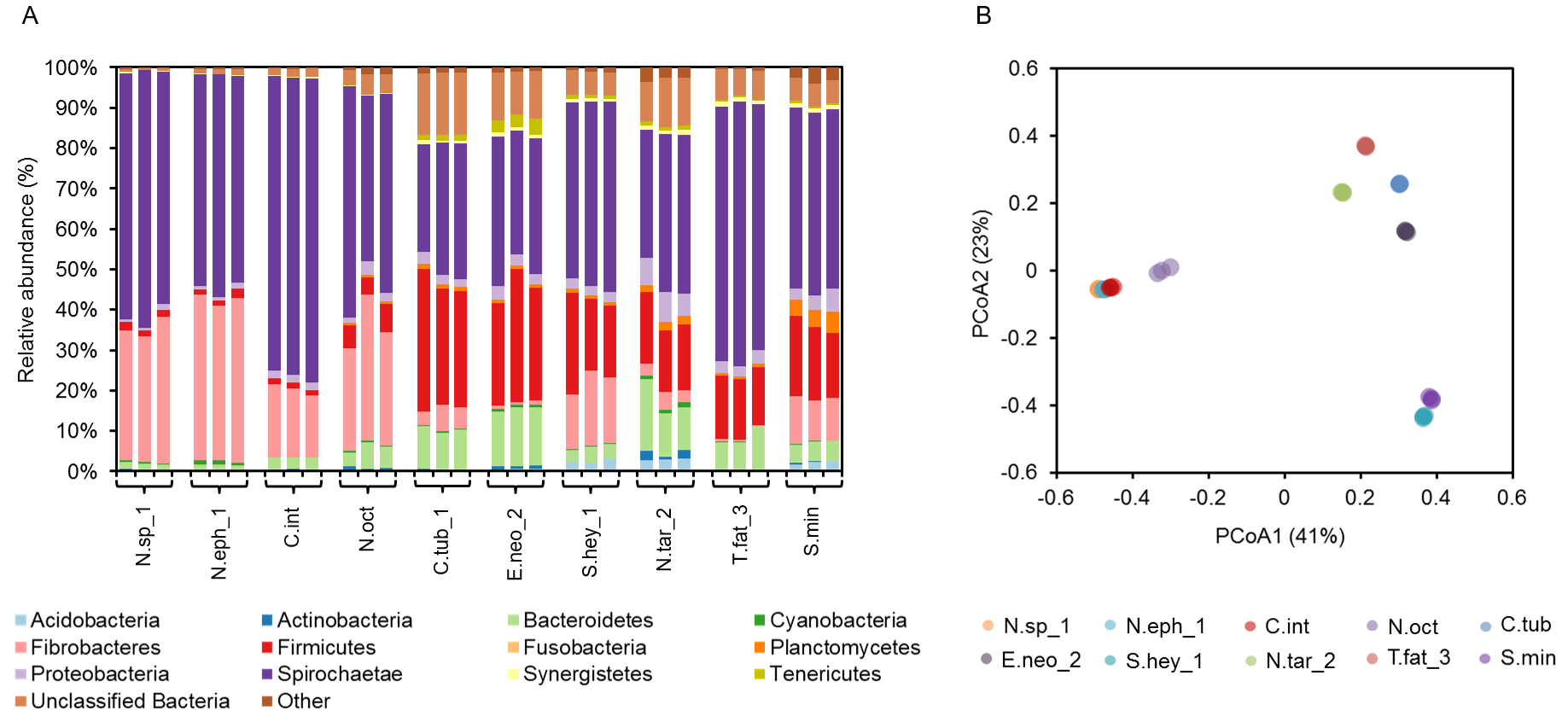


**Fig. S1** 16S rRNA gene amplicon sequencing results for triplicates, presented for 10 selected samples. PCoA ordination of the calculated Bray-Curtis dissimilarities in bacterial community structures at the OTU level showed close grouping of triplicates.


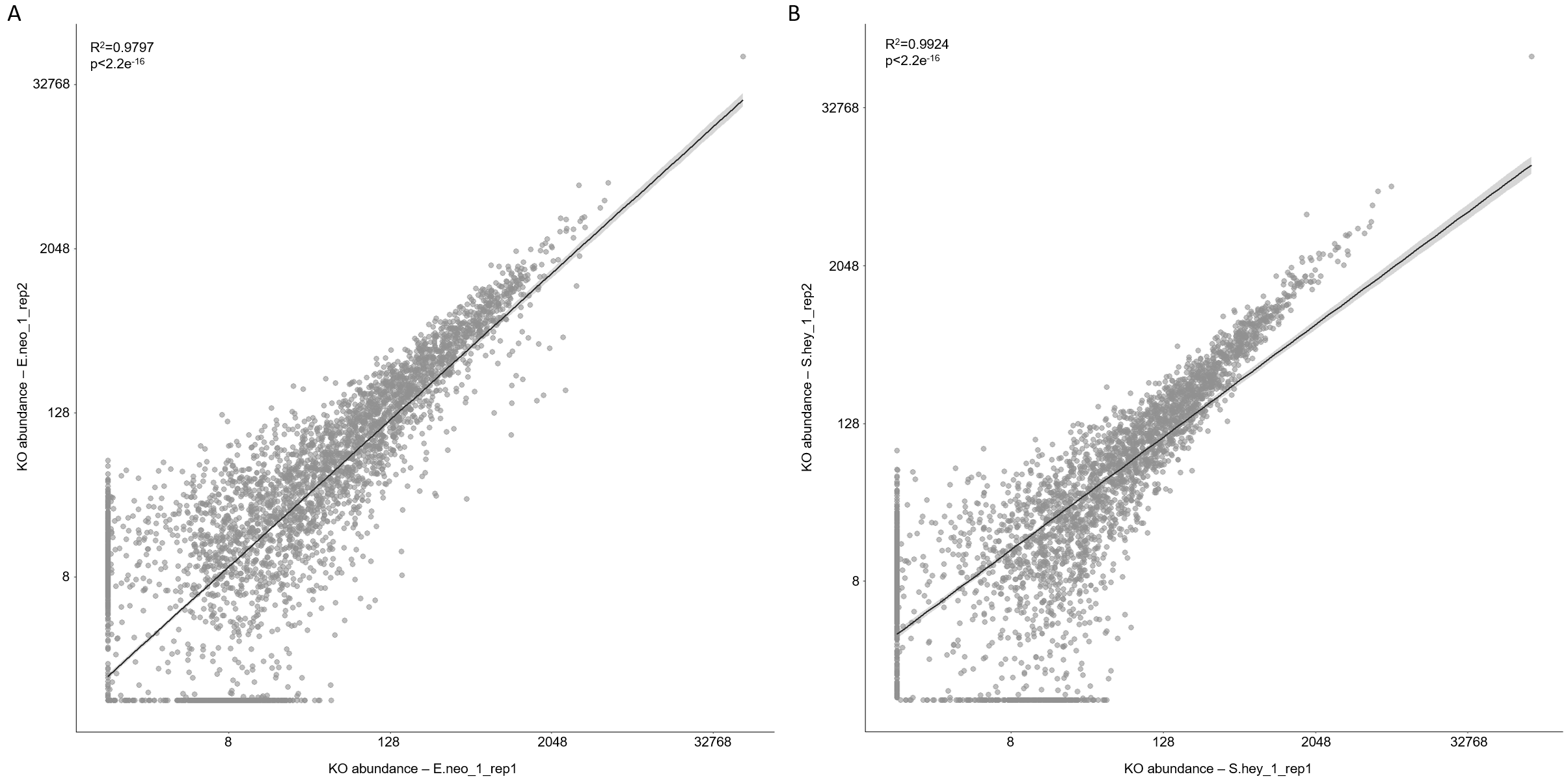


**Fig. S2** Duplicates (biological replicates) of metatranscriptomic libraries for two selected colonies (E.neo_1, S.hey_1). **A.** Cumulative abundance of all transcripts annotated to KEGG Ontology categories (KO abundance), for E.neo_1_rep1 *versus* E.neo_1_rep2 (R^2^=0.9797, p-value< 2.2e^-16^). **B.** Cumulative abundance of all transcripts annotated to KEGG Ontology categories (KO abundance), for S.hey_1_rep1 *versus* S.hey_1_rep2 (R^2^=0.9797, p-value< 2.2e^-16^).


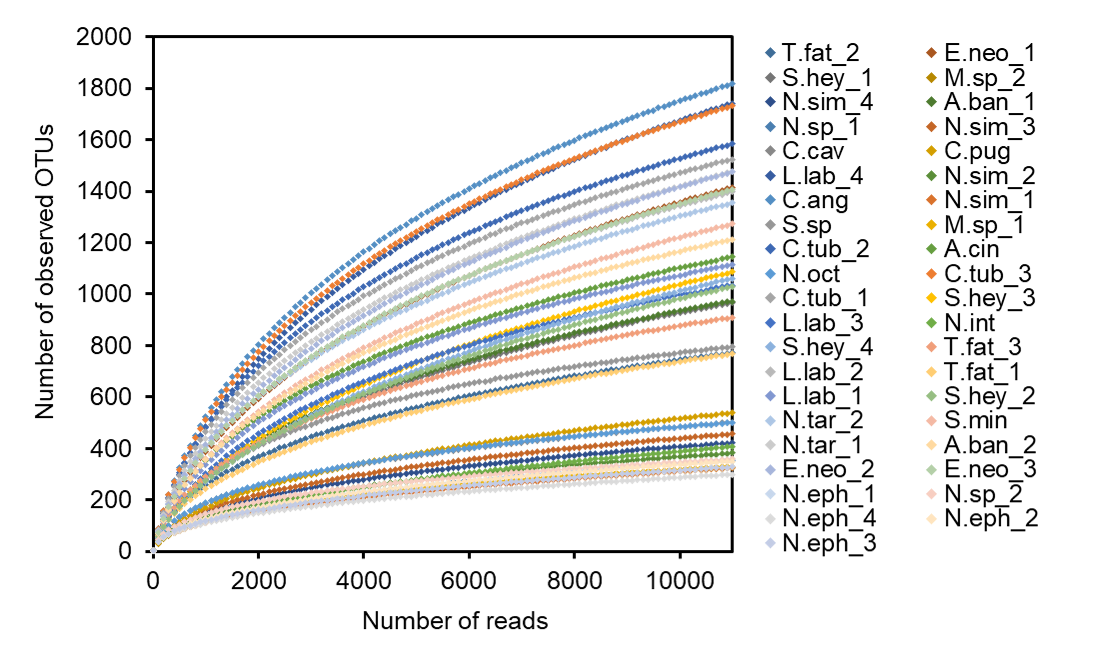


**Fig. S3** The observed richness estimator rarefaction curves based on high-throughput amplicon sequencing of 16S rRNA gene for 41 tested samples of termite gut bacteria.


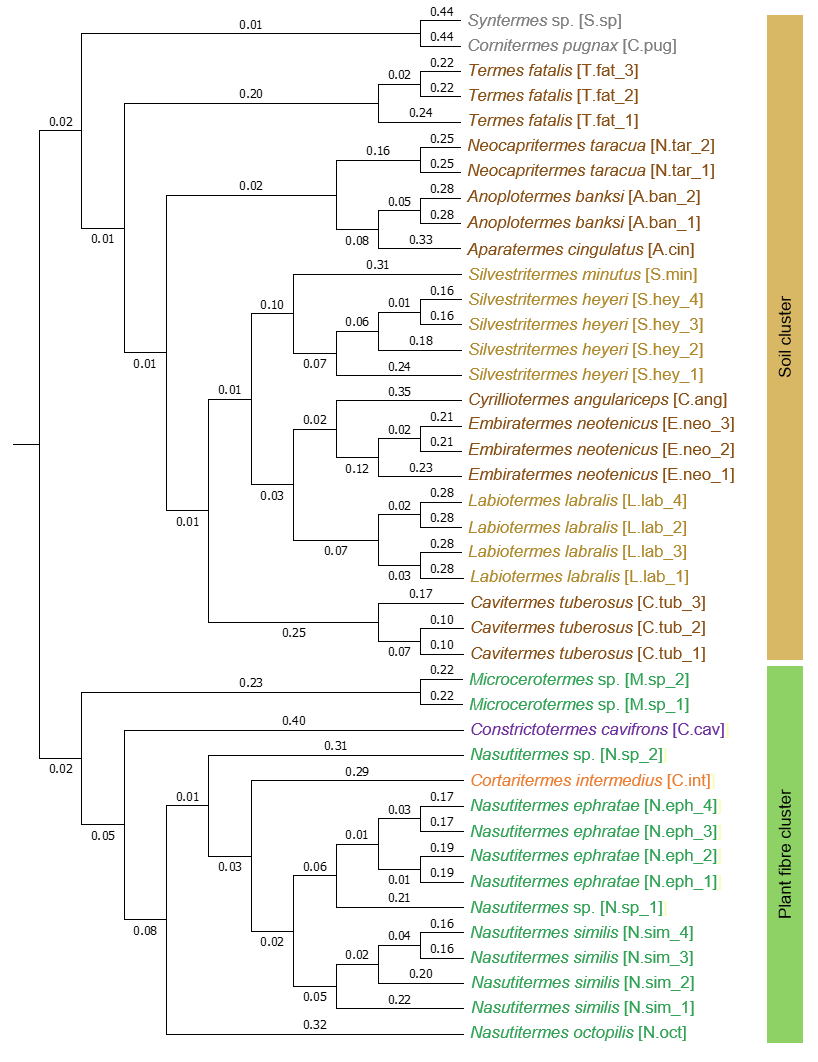


**Fig. S4** Tree based on the calculated Jaccard similarity in bacterial community membership, based on 16S rRNA gene amplicon sequencing; ANOSIM R for the two clusters was equal to 0.98 with p < 0.001.


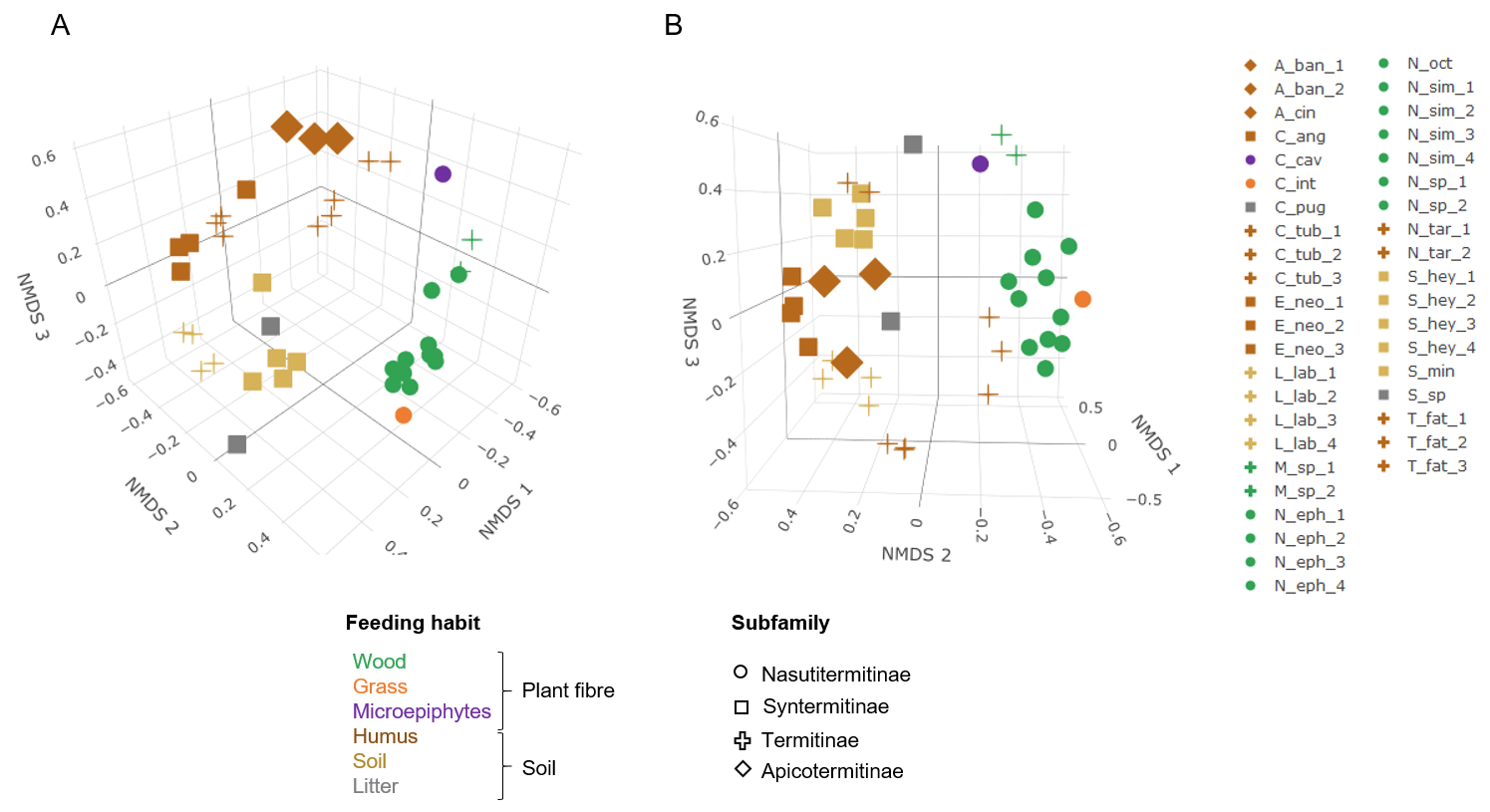


**Fig. S5** 3D-NMDS ordination of the calculated Bray-Curtis (A) and Jaccard (B) dissimilarities in bacterial community structures at the OTU level. Stress values: 0.21, R^2^=0.66 (Bray-Curtis) and 0.23, R^2^=0.63 (Jaccard).


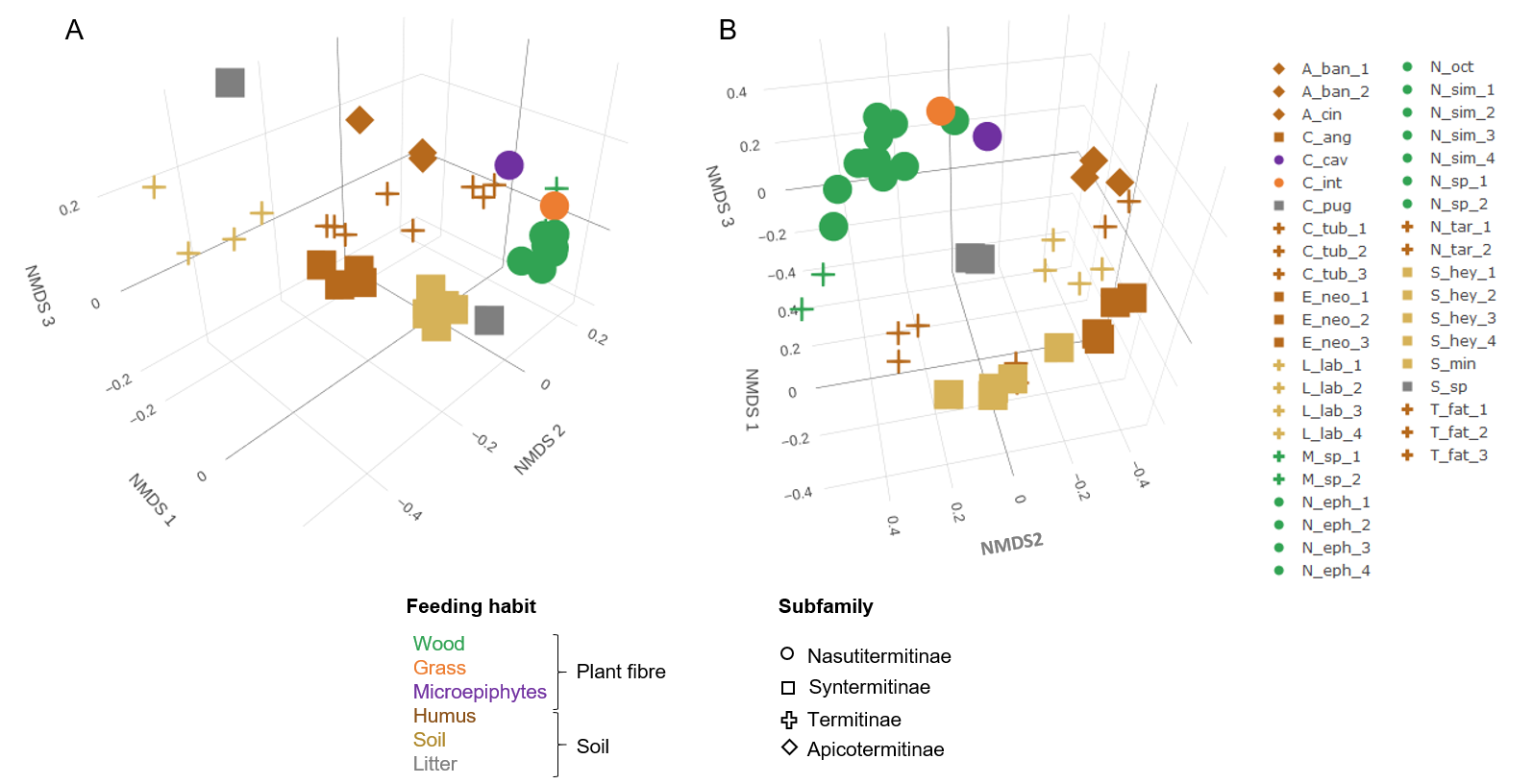


**Fig. S6** 3D-NMDS ordination of the weighted (A) and unweighted (B) UniFrac-calculated pairwise distance across all samples. Stress values: 0.09, R^2^=0,96 (weighted) and 0.21, R^2^=0.73 (unweighted).


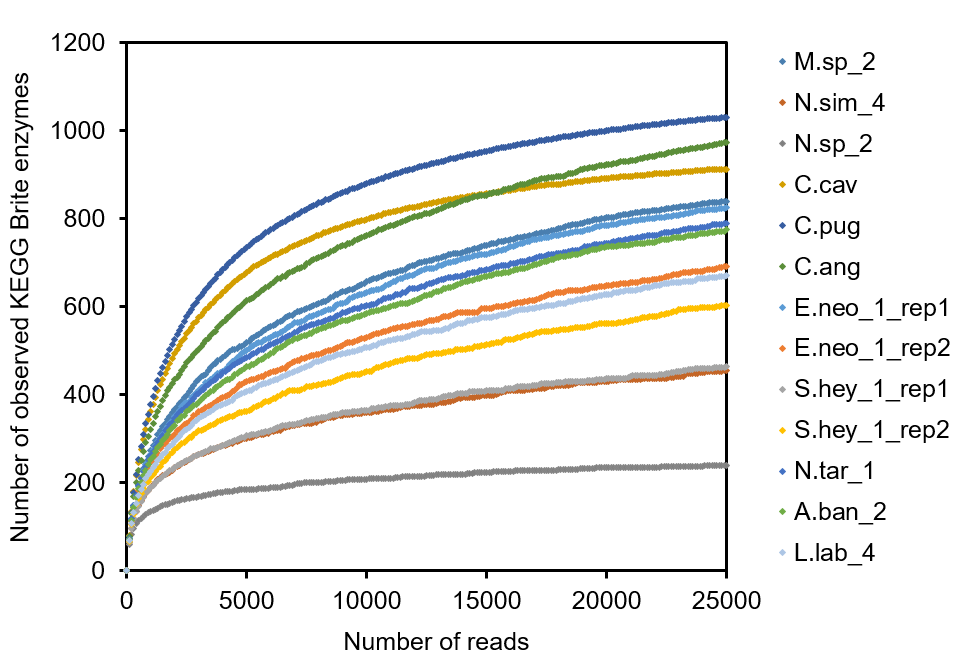


**Fig. S7** The calculated rarefaction curves of all the captured enzymatic annotations (reflected by the assigned KEGG BTITE enzyme numbers).


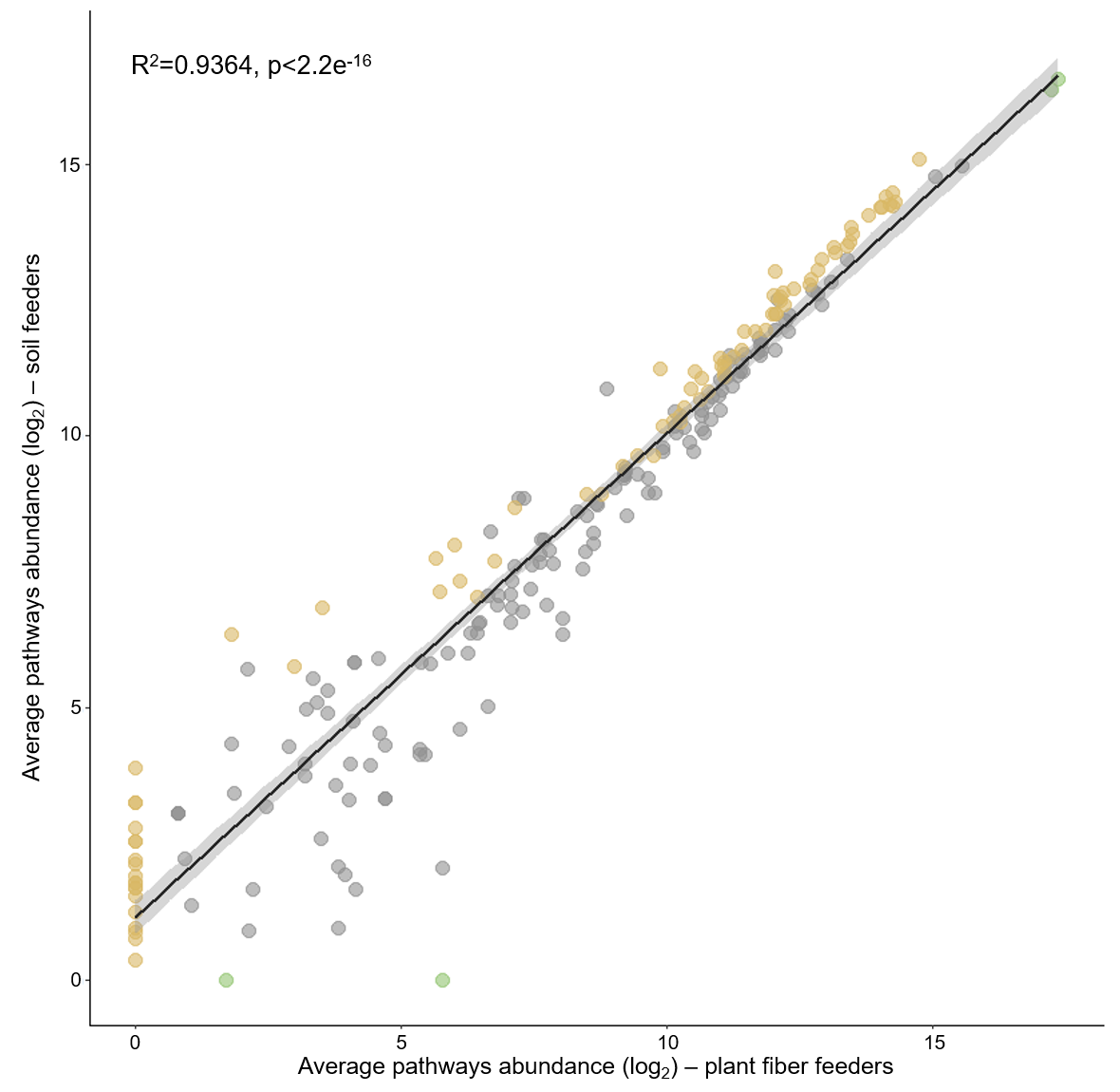


**Fig. S8** Average expression of pathways (cumulative abundance of transcripts assigned to given pathway) across prokaryotic microbiomes of plant fibre- and soil-feeding termites. Pathways enriched (LEfSE analysis) or present exclusively in plant fibre- or soil feeders are marked in green and brown colour, respectively. R^2^=0.9364; p-value <2.2e^-16^


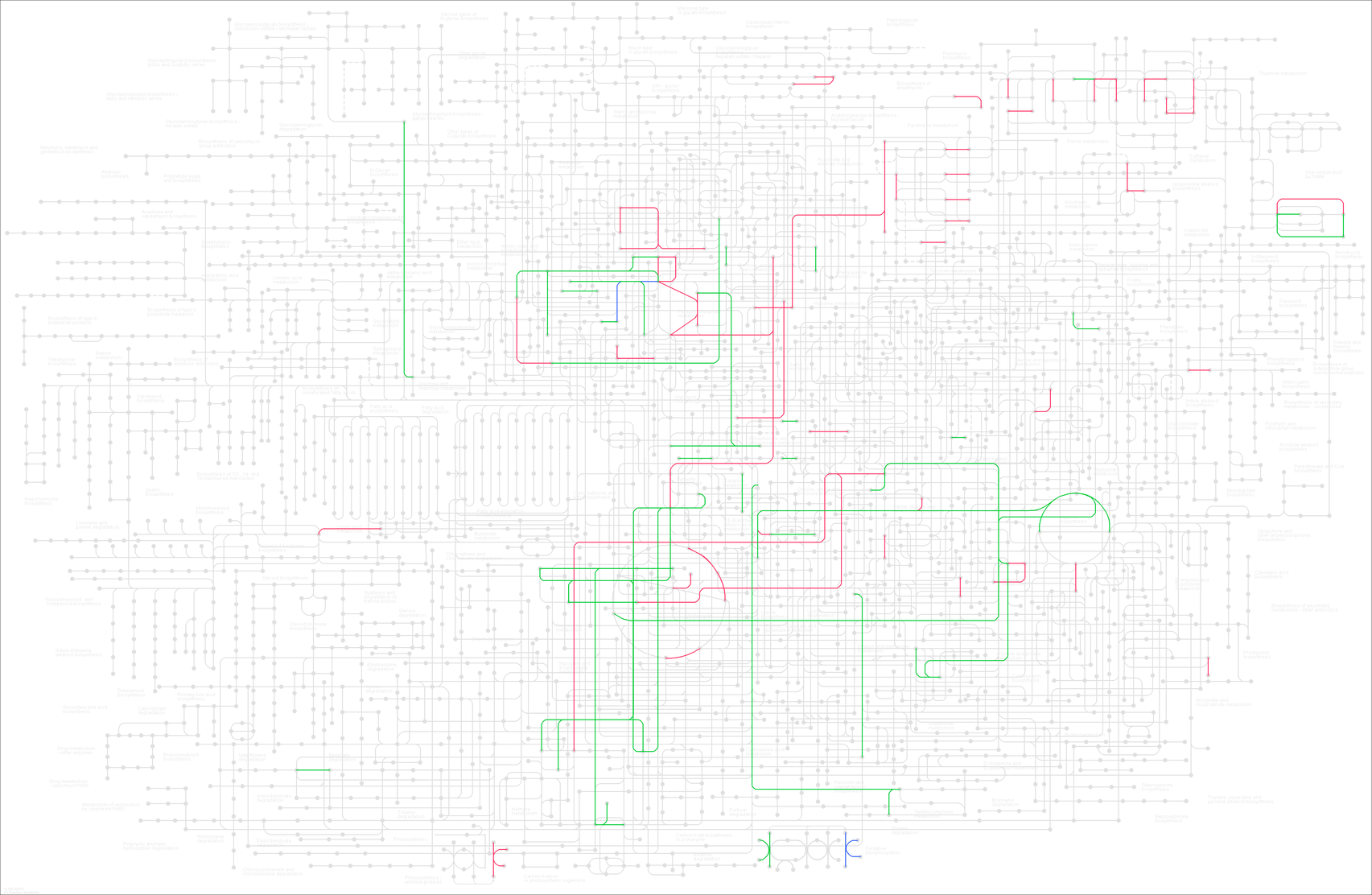


**Fig. S9** Illustration of the overrepresented KEGG Ontology categories showing low metabolic overlap between the two clusters in terms of cluster-specific functionalities. Functionalities specific to plant fibre- or soil-feeding termite cluster are marked in green and red, respectively. Overlapping functions are marked with blue.


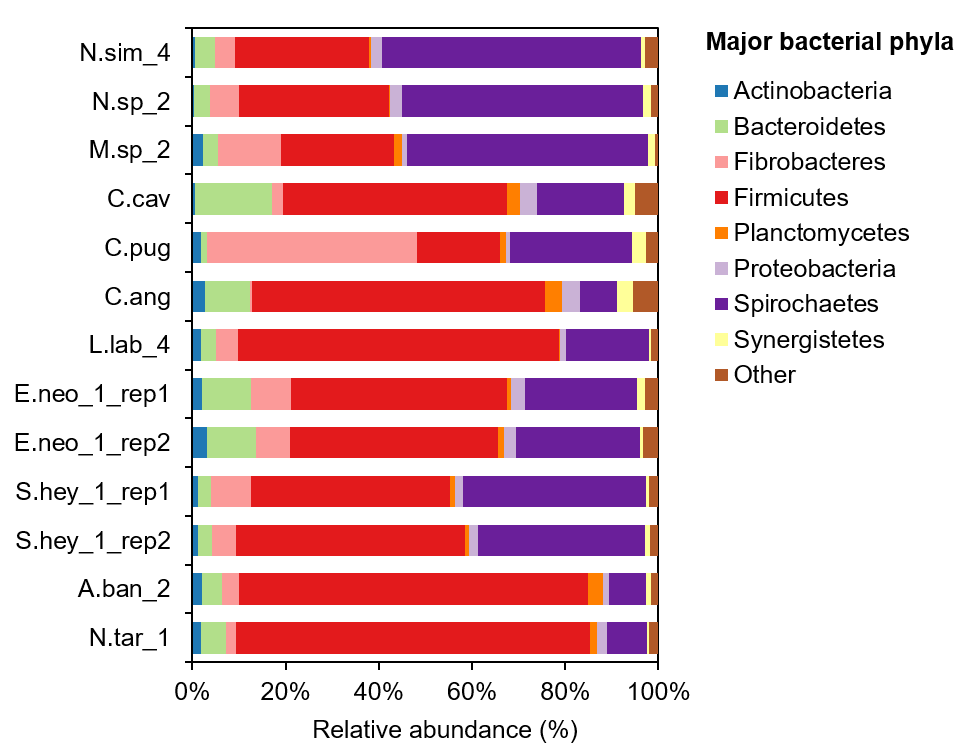


**Fig. S10** Taxonomic prediction of prokaryotic groups contributing the putative CAZymes expression in plant fibre- and soil-feeding termites.


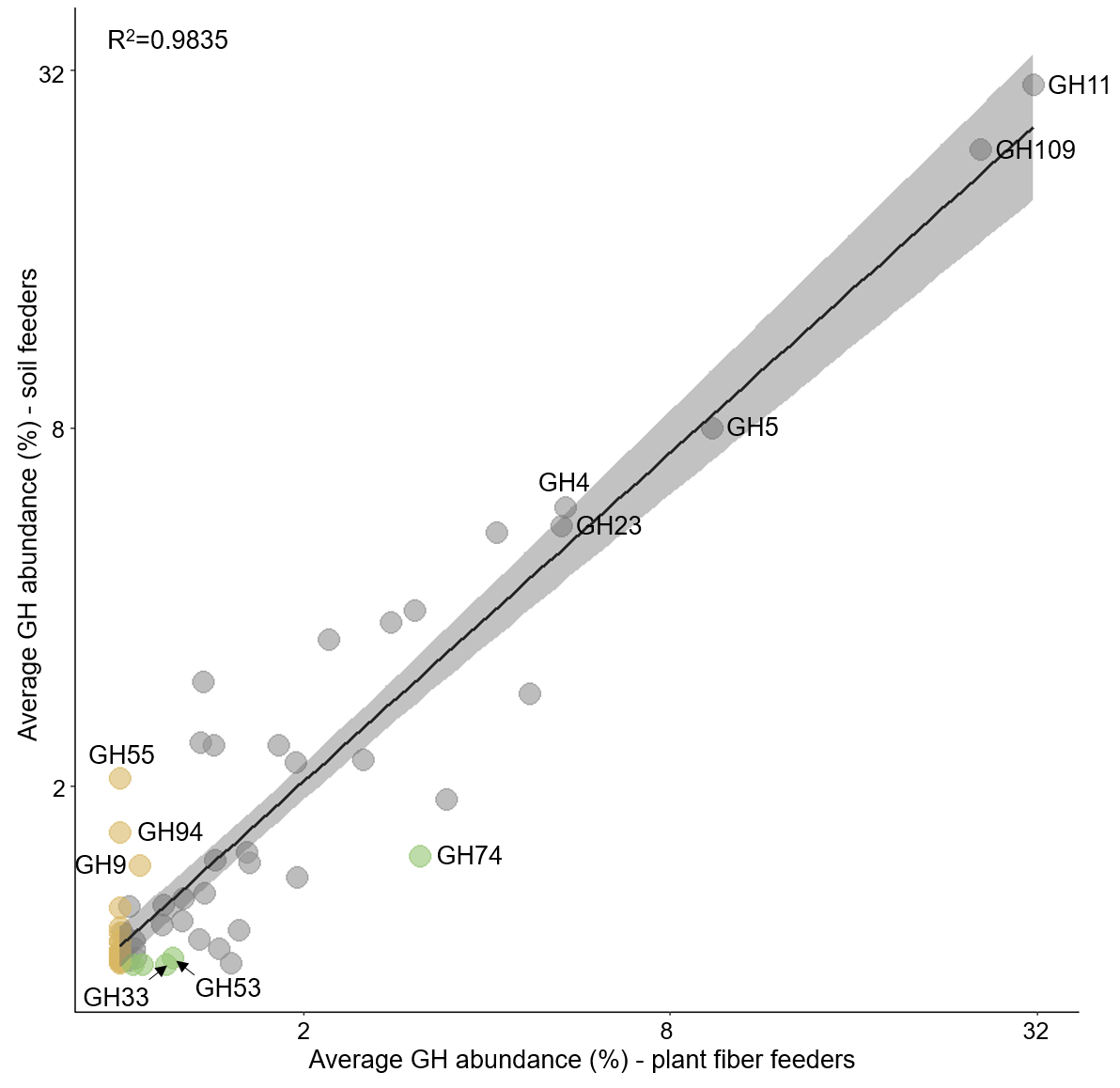


**Fig. S11** Average GH expression in prokaryotic gut microbiomes of plant fibre- and soil-feeding termites (results without application of the dbCAN tool threshold of e-value <10^−18^ and coverage >0.35); GHs enriched (LEfSE analysis) or present exclusively in plant fibre- or soil-feeding termite cluster are marked in green and brown colour, respectively.


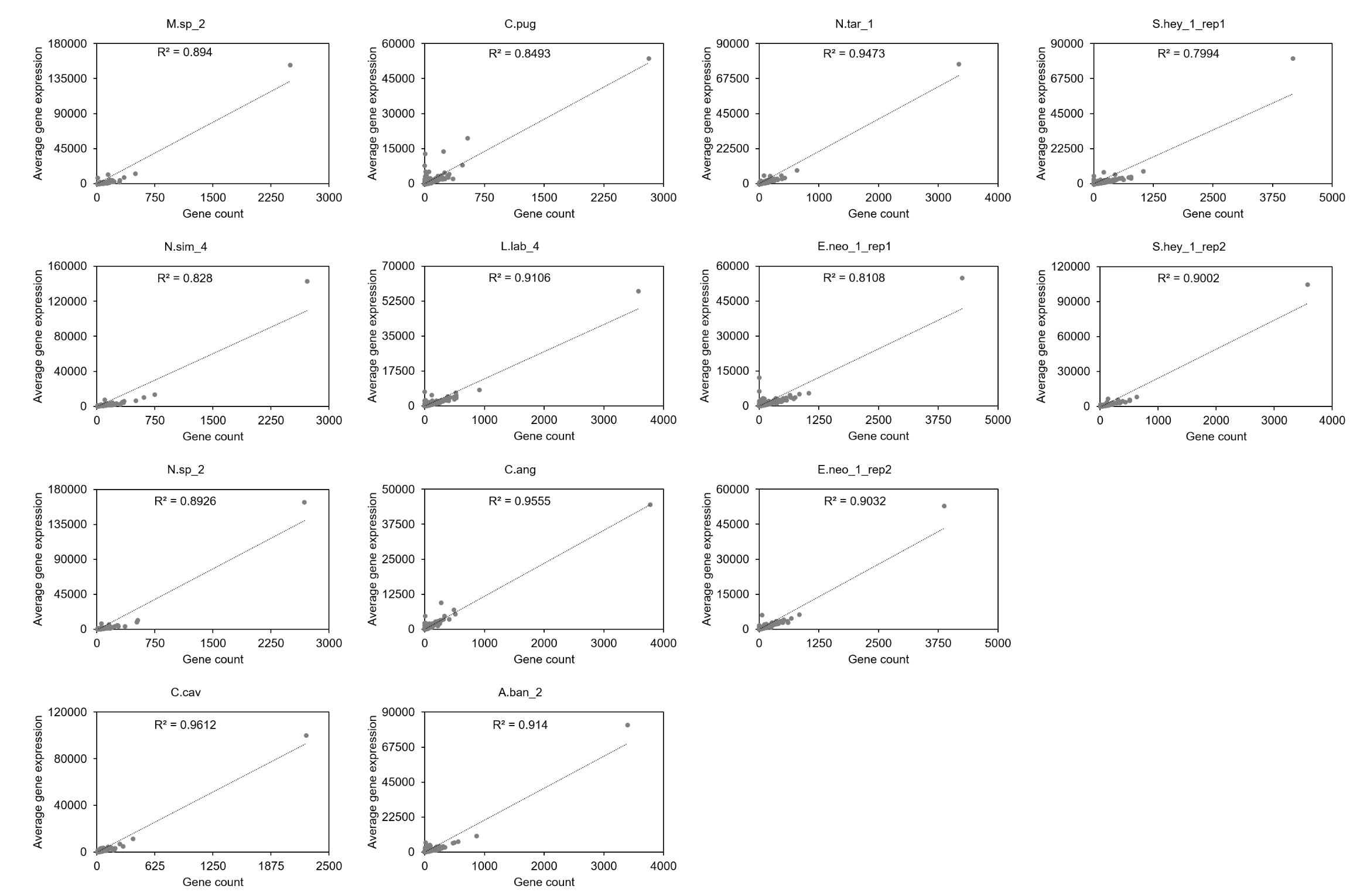


**Fig. S12** Correlation between the number of gene transcripts assigned to a gene category and its cumulative expression per sample.

**
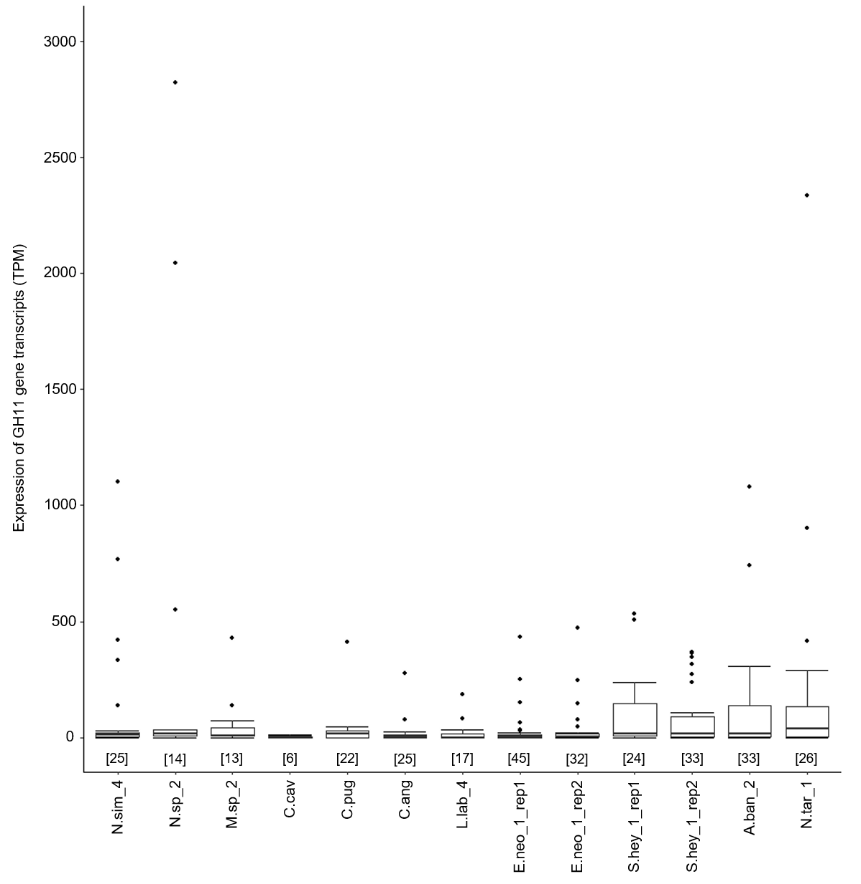
**

**Fig. S13** Expression of the gene transcripts assigned to GH11 CAZy family across all prokaryotic microbiomes. Number in the square bracket corresponds to the number of gene transcripts per each sample.
